# NIS-Seq enables cell-type-agnostic optical perturbation screening

**DOI:** 10.1038/s41587-024-02516-5

**Published:** 2024-12-19

**Authors:** Caroline I. Fandrey, Marius Jentzsch, Peter Konopka, Alexander Hoch, Katja Blumenstock, Afraa Zackria, Salie Maasewerd, Marta Lovotti, Dorothee J. Lapp, Florian N. Gohr, Piotr Suwara, Jędrzej Świeżewski, Lukas Rossnagel, Fabienne Gobs, Maia Cristodaro, Lina Muhandes, Rayk Behrendt, Martin C. Lam, Klaus J. Walgenbach, Tobias Bald, Florian I. Schmidt, Eicke Latz, Jonathan L. Schmid-Burgk

**Affiliations:** 1 Institute of Clinical Chemistry and Clinical Pharmacology, University and University Hospital Bonn, Bonn, Germany; 2 Institute of Innate Immunity, University and University Hospital Bonn, Bonn, Germany; 3 Department of Microbiology and Immunology, Peter Doherty Institute for Infection and Immunity, University of Melbourne, Melbourne, VIC Australia; 4 Appsilon, Warszawa, Poland; 5 Division of Plastic, Reconstructive and Aesthetic Surgery, Department of Surgery, University and University Hospital Bonn, Bonn, Germany; 6 Institute of Experimental Oncology, University and University Hospital Bonn, Bonn, Germany; 7 German Leibniz Centre for Rheumatism Research, Berlin, Germany

**Keywords:** Functional genomics, High-throughput screening, Sequencing

## Abstract

Optical pooled screening offers a broader-scale alternative to enrichment-based perturbation screening, using fluorescence microscopy to correlate phenotypes and perturbations across single cells. Previous methods work well in large, transcriptionally active cell lines, because they rely on cytosolic detection of endogenously expressed barcoded transcripts; however, they are limited by reliable cell segmentation, cytosol size, transcriptional activity and cell density. Nuclear In-Situ Sequencing (NIS-Seq) expands this technology by creating bright sequencing signals directly from nuclear genomic DNA to screen nucleated cells at high density and high library complexity. By inserting an inverted phage promoter downstream of the single guide RNA (sgRNA), many RNA copies of the sgRNA can be generated and sequenced independently of cellular transcription. In this study, we benchmarked NIS-Seq across eight cell types from two species and performed four genome-scale optical perturbation screens, identifying key players of inflammation-related cellular pathways. Finally, we performed a small-scale pooled optical screen in primary human macrophages from blood of healthy donors and demonstrated barcode identification in lentivirally transduced human skin tissue.

## Abstract (web summary)

An optical pooled screening method can correlate phenotypes and perturbations in any nucleated cell type.

## Main

Genetic perturbation screens aim to decipher the relationship between genotype and phenotype by ablating random genes in a pool of cells. Perturbation screens are commonly based on enrichment of cells with a phenotype of interest followed by next-generation sequencing (NGS) of the perturbagens, such as CRISPR single guide RNAs (sgRNAs)^1,2^ or gene trap insertions in haploid cells^3^. Enrichment of specific perturbagens allows the biological function of targeted genes to be determined. Although enrichment is commonly achieved through cell growth or fluorescence-activated cell sorting (FACS)^4,5^, more sophisticated screening approaches combine perturbation screens with single-cell molecular profiling via mass cytometry or RNA sequencing^6–8^. These methods acquire high-dimensional single-cell information but are incapable of monitoring dynamic processes.

With arrayed CRISPR screening, perturbed cells are physically separated into compartments containing cells with largely homogeneous genotypes. This allows analysis of cells using complex assays, such as fluorescence microscopy^9^, monitoring cell physiology in a spatially and temporally resolved manner. However, arrayed screens are laborious and prone to experimental inconsistencies between compartments, so they are mostly feasible for small targeted perturbagen libraries.

Optical pooled perturbation screening addresses these limitations by accessing the bandwidth of phenotypes that can be observed by fluorescence microscopy while working with a complex pool of cells^10^. Currently, several approaches to optical pooled perturbation screening have been developed. For optical-based enrichment, cells with a phenotype of interest are identified by microscopy and are marked by light-based conversion of a photoactivatable fluorescent protein. Subsequently, cells are dissociated; marked cells are enriched by FACS; and perturbagens are analyzed by deep sequencing^11^. Similarly, image-based cell sorting^12^ or laser microdissection^13^ can be used to isolate cells displaying a microscopic phenotype of interest for subsequent perturbagen identification, albeit at limited optical resolution. Instead of enriching for a population of cells for lysis and sequencing, Feldman et al.^10^ developed a method for microscopic perturbagen identification based on in situ sequencing of barcodes contained in cellular mRNAs, using signal amplification by rolling circle amplification (RCA). Using this method, both the phenotype and the genotype are recorded from individual cells using fluorescence microscopy, allowing categorization of cellular phenotypes post hoc^14^. This method was recently adapted to screen genome-scale perturbation libraries for pre-defined^15^ and multidimensional phenotypes^16,17^.

Although in situ sequencing-based screening has the advantage of post hoc genetic dissection of multidimensional phenotypes, current methods still bear two major limitations. First, because sequencing is dependent on mRNA molecules in the cytosol, cells with low transcriptional activity or small volume generate lower signal, precluding some relevant cell types from optical pooled screening. Furthermore, cells have to be permeabilized for in situ sequencing. Thus, a loss of precise cell–cell boundaries can interfere with assignment of cytosolic spots to nuclei at high cell densities and complex barcode distributions.

To extend pooled optical screening toward any nucleated cell type, we developed Nuclear In-Situ Sequencing (NIS-Seq). After phenotyping live cells and fixation, the reverse-complement sequences of perturbation gRNAs are locally transcribed from genomic DNA using T7 polymerase by an adapted Zombie protocol^18^. Nuclear clusters of RNA are efficiently sequenced by padlock-based three-color in situ sequencing. NIS-Seq enables unambiguously assigning bright signal clusters to nuclei independent of cell size, type or transcriptional activity. Using this technology, we performed optical pooled perturbation screens in primary human cells and cell lines and demonstrated barcode identification in human skin tissue.

## Results

### Cell-type-agnostic optical barcode identification

**Fig. 1:**
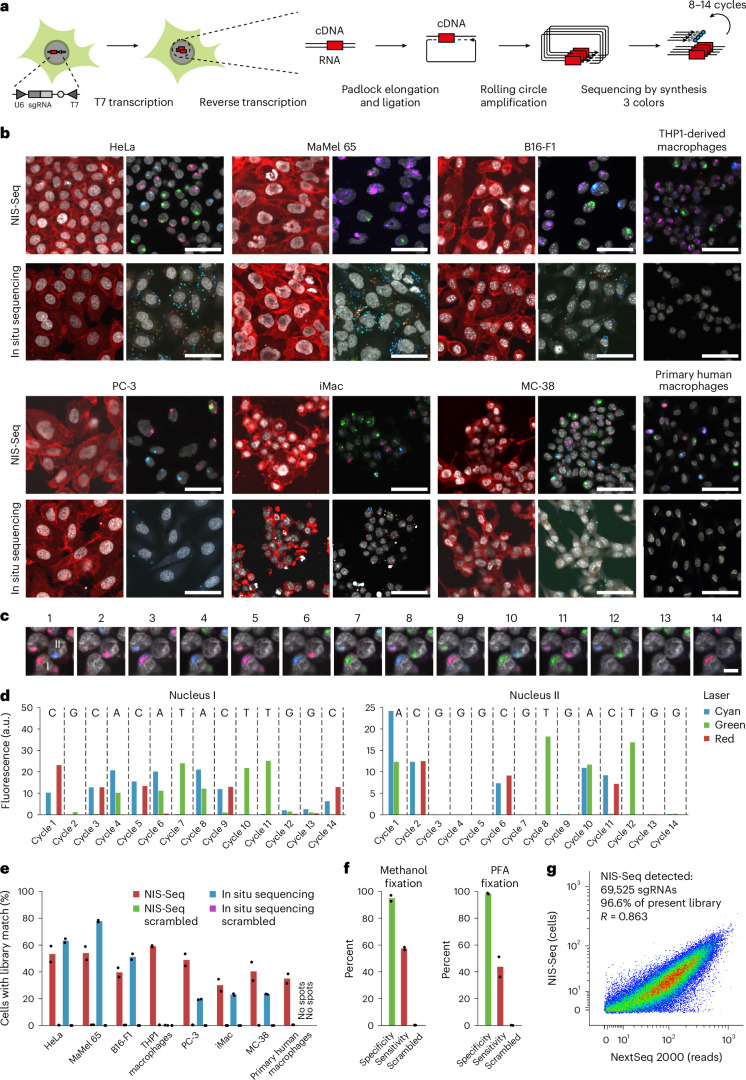
NIS-Seq enables optical barcode identification in nucleated cells. **a**, Outline of NIS-Seq reaction steps, adding a T7 transcription step to previously established in situ sequencing of barcoded mRNA^10^. **b**, First-cycle NIS-Seq imaging results in comparison to cytosolic in situ sequencing results obtained across eight cell types. Live cells were stained for nuclei (gray) and membrane (red) before fixation (left panels). Scale bar, 50 µm. Representative images from two experimental replicates are shown. **c**, Raw images of 14 cycles of NIS-Seq barcode sequencing from THP1 cells. Nuclear staining was performed at cycles 1, 4, 7, 10 and 13. Scale bar, 10 µm. Representative data from four experimental replicates are shown. **d**, Quantitative spot intensities obtained from THP1 cell nuclei I and II highlighted in **c**. Indicated on top is the base calling result, matching two members of the pooled lentiviral library used. **e**, Fraction of cells mapping to known library member sequences. Scrambled controls indicate analysis results using a permutated reference library with equal base distribution. Dots indicate analysis results from two halves of a 0.56-cm^2^ surface. Two rows of edge tiles were excluded from analysis to avoid empty or distorted images. **f**, Specificity and sensitivity of NIS-Seq was quantified using a mixed population of cells either expressing GFP or containing NIS-Seq-compatible genomic insertions. The standard NIS-Seq protocol was compared to a modified version using PFA fixation and subsequent de-crosslinking (Methods). Dots indicate analysis results from two independent wells. **g**, Library coverage in transduced THP1 macrophages, measured by PCR-based NGS and NIS-Seq. Each library member covered in PCR-based sequencing is represented by one dot; dropping out sgRNAs—for example, those targeting essential genes—are not shown. Jitter was added to values to visualize spot density at low integer values.

Based on adapting two previously published methods^10,18^, we developed NIS-Seq to enable cell-type-agnostic high-density optical CRISPR screening. Although published in situ sequencing detects barcodes from RNA polymerase II–expressed mRNAs in the cytosol, NIS-Seq uses T7 in vitro transcription (IVT) to generate multiple RNA copies in the nucleus (Fig. 1a and Supplementary Fig. 1a). After subsequent reverse transcription, padlock elongation, ligation and RCA, sgRNAs are identified by up to 14 cycles of sequencing-by-synthesis using three excitation wavelengths (Fig. 1a). To test this approach, we first developed -Seq-compatible lentiviral vector that enables high genome editing efficiencies. We compared different vector designs across three genomic target sites in HeLa–Cas9 cells. Genome editing efficiencies were determined by NGS after puromycin selection and Cas9 induction^19^, confirming that inserting an inverted T7 promoter after the polymerase III terminator of an sgRNA expression cassette mirrors efficiencies of state-of-the-art lentiviral CRISPR vectors (Supplementary Fig. 1b). This vector was equipped with a well-established human genome-scale sgRNA library^20^ and used to produce pools of lentiviral particles.

Not relying on transcriptional activity, cytosolic volume or precise cell boundaries, NIS-Seq should enable unambiguous assignment of library members to cells even at high cell densities as required for genome-scale screening. We tested NIS-Seq and standard in situ sequencing across a panel of eight cell types from two species. NIS-Seq yielded bright-colored spots in all cell types tested, including THP1-derived and primary human macrophages, whereas the previously published in situ sequencing protocol did not yield visible signal in the latter cell types (Fig. 1b). Over the course of 14 cycles of sequencing a library of THP1 cells, NIS-Seq generated distinct altering color combinations in individual nuclei (Fig. 1c), which can be translated to known sequences of library members (Fig. 1d). Analyzing 14 cycles of NIS-Seq imaging data, NIS-Seq assigned cells to library members consistently across eight cell types, whereas lower and more cell-type-dependent mapping was observed with cytosolic in situ sequencing; mapping rates were blunted using a scrambled reference library with both methods (Fig. 1e).

For accurate mapping of live-cell phenotyping data to NIS-Seq data using different objectives and high cell densities, we optimized a cross-correlation-based search algorithm that can compensate small movements and distortions of cells and surfaces during live-cell imaging, assigning nuclei between imaging modalities with high confidence (Supplementary Fig. 1c). To test the quality of nuclear assignments as well as to verify that NIS-Seq signals are detected only in cells bearing a NIS-Seq construct, we mixed GFP-expressing HeLa cells with NIS-Seq library transduced HeLa cells and compared the GFP signal in live cells before fixation to the NIS-Seq library mapping result after fixation. NIS-Seq and nuclear assignments proved to be highly specific across two fixation protocols, which render the method compatible with antibody staining (Fig. 1f). Finally, quantifying the representation of individual library members by NIS-Seq and polymerase chain reaction (PCR)-based NGS in a subsampled pool of THP1-derived macrophages confirmed a high correlation between methods across 69,525 library members detected in the cells by NGS (Fig. 1g).

### Dissection of immune receptor signaling in HeLa cells

**Fig. 2:**
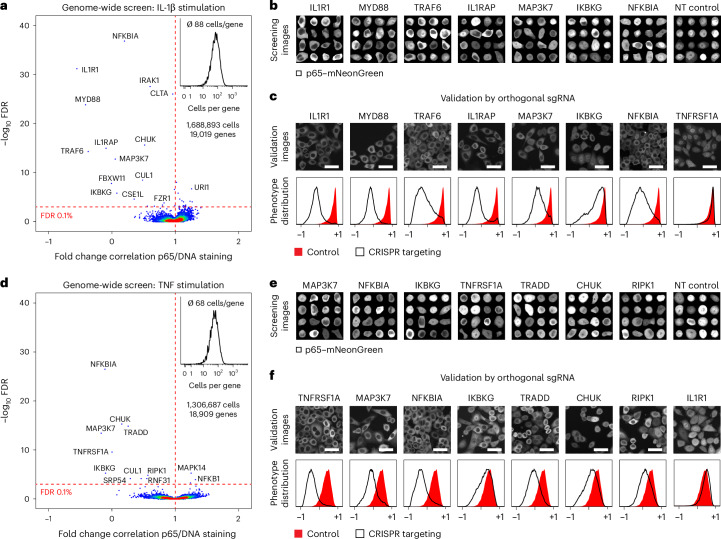
Genome-scale optical perturbation screening for mediators of NF-κB activation. **a**, Genome-wide NIS-Seq perturbation screening in HeLa–Cas9–p65–mNeonGreen cells stimulated with IL-1β. Fold changes are calculated based on the mean pixel-wise Pearson correlation between mNeonGreen and nuclear staining signals across cells with the same targeted gene versus non-targeting (NT) control cells. Deviation of their correlation value distributions was tested using a two-sided Wilcoxon–Mann–Whitney test followed by the Benjamini–Hochberg procedure. **b**, Collages of cellular images from **a** mapped to perturbed genes indicated. Shown is the mNeonGreen signal. **c**, Arrayed hit validation in HeLa–Cas9–p65–mNeonGreen cells using alternative sgRNA sequences from the TKOv3. Top panel, exemplary mNeonGreen images of IL-1β-stimulated cells. Bottom panel, distribution of activation states, quantified by pixel-wise Pearson correlation between mNeonGreen and nuclear staining signals. Scale bar, 50 µm. Representative data from two experimental replicates are shown. **d**, Genome-wide NIS-Seq perturbation screening in HeLa–Cas9–p65–mNeonGreen cells stimulated with TNF. Fold changes were calculated based on the mean pixel-wise Pearson correlation between mNeonGreen and nuclear staining signals across cells with the same targeted gene versus NT control cells. Deviation of their correlation value distributions was tested using a two-sided Wilcoxon–Mann–Whitney test followed by the Benjamini–Hochberg procedure. **e**, Collages of cellular images from **d** mapped to perturbed genes indicated. Shown is the mNeonGreen signal. **f**, Arrayed hit validation in HeLa–Cas9–p65–mNeonGreen cells using alternative sgRNA sequences from the TKOv3. Top panel, exemplary mNeonGreen images of TNF-stimulated cells. Bottom panel, distribution of activation states, quantified by pixel-wise Pearson correlation between mNeonGreen and nuclear staining signals. Scale bar, 50 µm. Representative data from two experimental replicates are shown.

To demonstrate genome-scale screening applications of NIS-Seq, we targeted HeLa cells to investigate genes involved in the activation of nuclear factor (NF)-κB, a family of five members of inducible transcription factors functioning as homodimers or heterodimers^21^. In an inactive state, NF-κB dimers are retained in the cytosol by inhibitory proteins, such as IκB. Upon activation of the pathway by external stimuli, phosphorylation of the Iκk complex leads to ubiquitination and proteasomal degradation of IκB, allowing dimeric NF-κB to shuttle into the nucleus^21^. To study genes involved in this process, we used a previously established translocation assay based on HeLa cells carrying a fluorescent p65–mNeonGreen reporter^10^. To elucidate pathway members, we performed two replicate screens using 76,441 sgRNAs targeting human protein-coding genes as well as 1,000 non-targeting control guides. HeLa cells were stimulated with interleukin 1β (IL-1β) or tumor necrosis factor (TNF) to target two different pathways of NF-κB activation. After live-cell phenotyping, the sgRNA identity present in each cell was determined by NIS-Seq. Mapping of phenotype and NIS-Seq nuclei resulted in more than 18,000 genes covered by at least 10 cells for each stimulus (Fig. 2a,d; detailed statistics of all NIS-Seq screens in Supplementary Table 2). Nuclear translocation of p65 was quantified as the pixel-wise Pearson correlation coefficient between p65–mNeonGreen and nuclear staining images. Genes with altered mean nuclear translocation across targeted cells were identified by false discovery rate (FDR)-corrected statistical testing and corresponded to the receptors *IL1R1* and *TNFRSF1A* as well as multiple known downstream pathway members, such as *TRAF6*, *CHUK* and *IKBKG*, which were confirmed by single-cell collages of targeted cells retrieved from pooled imaging data (Fig. 2b,e). Strong outlier genes were furthermore confirmed using orthogonal sgRNAs from the Toronto KnockOut CRISPR library v3 (TKOv3) (ref. ^22^) (Fig. 2c,f). Screening hits largely match the results of a targeted optical pooled screen^10^ (Supplementary Fig. 1d).

### Optical screening for inflammasome activation in macrophages

**Fig. 3:**
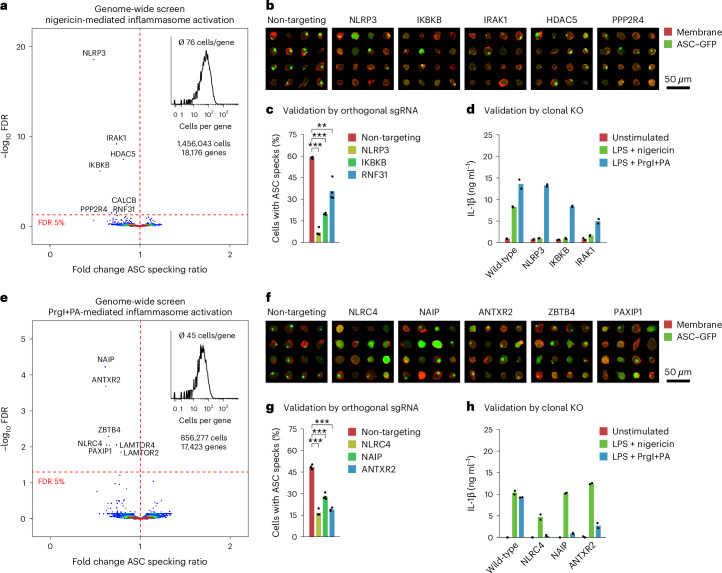
Genome-scale optical perturbation screening in THP1-derived macrophages for inflammasome activation. **a**, Genome-wide NIS-Seq perturbation screening in THP1–Cas9–ASC–GFP–CASP1/8^DKO^ cells stimulated with nigericin, which is a known trigger of the NLRP3 inflammasome. Fold changes were calculated based on the high-frequency filtered GFP signal relative to the overall GFP signal per cell across cells with the same targeted gene versus non-targeting control cells. Deviation of gene-wise value distributions from controls was tested using a two-sided Wilcoxon–Mann–Whitney test followed by the Benjamini–Hochberg procedure. **b**, Collages of cellular images from **a** mapped to perturbed genes indicated. Shown are membrane stain (red) and ASC–GFP (green) signals. **c**, Arrayed hit validation in THP1–Cas9–ASC–GFP cells using alternative sgRNA sequences from the TKOv3. Shown are fractions of cells with an ASC speck upon nigericin stimulation from four independent replicate viral transductions. **P < 0.01 and ***P < 0.001, two-sided *t*-test. **d**, Cytokine secretion in response to two inflammasome triggers in wild-type or clonal gene-deficient THP1–ASC–GFP cells, measured by IL-1β ELISA. Shown is the mean of two technical replicate measurements from two biological replicates. **e**, Genome-wide NIS-Seq perturbation screening in THP1–Cas9–ASC–GFP–CASP1/8^DKO^ cells stimulated with PrgI+PA, which is a known trigger of the NLRC4 inflammasome. Fold changes were calculated based on the high-frequency filtered GFP signal relative to the overall GFP signal per cell across cells with the same targeted gene versus non-targeting control cells. Deviation of gene-wise value distributions from controls was tested using a two-sided Wilcoxon–Mann–Whitney test followed by the Benjamini–Hochberg procedure. **f**, Collages of cellular images from **e** mapped to perturbed genes indicated. Shown are membrane stain (red) and ASC-GFP (green) signals. **g**, Arrayed hit validation in THP1–Cas9–ASC–GFP cells using alternative sgRNA sequences from the TKOv3. Shown are fractions of cells with an ASC speck upon PrgI+PA stimulation from four independent replicate viral transductions. ***P < 0.001, two-sided *t*-test. **h**, Cytokine secretion in response to two inflammasome triggers in wild-type or clonal gene-deficient THP1–ASC–GFP cells, measured by IL-1β ELISA. Shown is the mean of three technical replicate measurements from two biological replicates.

NLRP3 inflammasomes are megadalton protein complexes that assemble in response to danger-associated and pathogen-associated molecular patterns in macrophages, leading to rapid IL-1β and IL-18 cytokine release as well as a rapid form of programmed cell death termed pyroptosis. Even though this pathway is critically involved in a multitude of age-related diseases, no genetic screening has been performed in human cells to systematically identify genetic components involved in physiological NLRP3 inflammasome assembly. Using NIS-Seq, we screened a genome-scale CRISPR knockout library of THP1 monocyte-derived macrophages stimulated with the ionophore nigericin as well as with the NLRC4 activator PrgI+PA. The cells used were deficient in Caspase-1 and Caspase-8 to avoid pyroptotic cell death downstream of early steps of inflammasome assembly. An ASC–GFP reporter enabled monitoring inflammasome assembly in live cells. Inflammasome activation was quantified in individual cells by acquiring z-stacks and calculating the ratio of overall GFP intensity to high-frequency filtered GFP intensity, the latter originating from smaller objects such as ASC specks. FDR-corrected statistical testing highlighted genes whose single-cell distribution of ASC specking ratios differed from the distribution of non-targeting control cells. We confirmed *NLRP3* and *IKBKB* as the most critical pathway member genes for NLRP3 activation^23^, whereas ablation of *IRAK1*, *HDAC5*, *CALCB*, *PPP2R4* and *RNF31* resulted in intermediate degrees of pathway dysfunction (Fig. 3a). Analogously, we confirmed *NLRC4*, its upstream receptor *NAIP* and the anthrax toxin receptor 2 (encoded by *ANTXR2)* to be required for PrgI+PA-mediated inflammasome activation (Fig. 3e), with additional genes *ZBTB4*, *PAXIP1* and two members of the *LAMTOR* family to be involved in inflammasome activation. Aggregated images of live cells assigned to hit genes confirmed a reduction in ASC specking as compared to control cells (Fig. 3b,f). Independent validation of hit genes by lentiviral expression of orthogonal guide RNAs from the TKO library confirmed reduced ASC specking in response to nigericin (Fig. 3c) and PrgI+PA (Fig. 3g), which was reflected in cytokine secretion levels in clonal knockout cell lines of hits (Fig. 3d,h).

### Dissection of inflammasome activation in primary macrophages

**Fig. 4:**
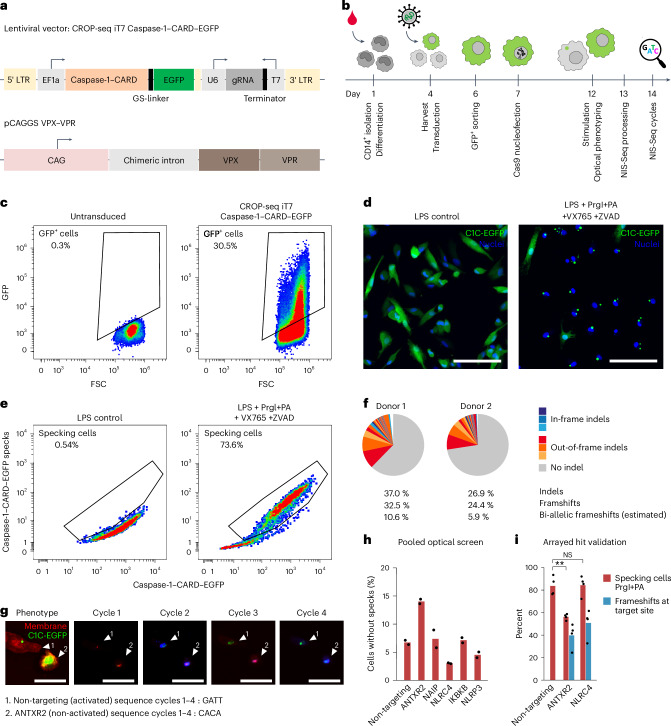
NIS-Seq perturbation screening in primary human macrophages. **a**, Lentiviral vector co-expressing an sgRNA and C1C–EGFP for monitoring inflammasome activation (top) and a VPX–VPR expression plasmid for efficient lentiviral transduction of primary human macrophages (bottom). **b**, Outline of NIS-Seq perturbation screening in primary human macrophages. **c**, EGFP expression in primary human M-CSF macrophages transduced with CROPseq-iT7 C1C–EGFP and analyzed by FACS. FSC, forward scatter. **d**, Confocal imaging of C1C–EGFP localization in primary human macrophages. Cells were primed with 20 ng ml^−1^ LPS for 3 h and activated after 30 min of caspase inhibition (40 µM VX-765 and 50 µM Z-VAD) by 1 h of stimulation with 1 µg ml^−1^ PA and 10 ng ml^−1^ PrgI. Nuclei were stained with Hoechst 33342 (blue). Scale bars, 100 µm. Representative data from two experimental replicates are shown. **e**, Image analysis results of FACS-sorted EGFP^+^ cells stimulated as in **c**. **f**, Genome editing efficiencies in primary human macrophages transduced with the lentiviral vector depicted in **a** and electroporated with SpCas9 protein. Genomic editing rates were quantified by NGS in cells from two healthy donors. **g**, Reporter activation and four cycles of NIS-Seq in primary human macrophages after stimulation as described in **d**. Highlighted are two cells assigned to either a non-targeting control or an *ANTXR2*-targeting sgRNA by NIS-Seq. Scale bars, 50 μm. Representative data from two biological replicates are shown. **h**, Pooled optical perturbation screen in primary human macrophages. Shown are relative fractions of cells with blunted reporter activation for each of five perturbed genes targeted by four sgRNAs each or eight non-targeting control sgRNAs. Data points indicate results from two independent screening wells, each containing a mixture of macrophages from two healthy donors. In total, 2,663 macrophage cells were mapped to both a library sgRNA and a phenotype. **i**, Arrayed validation using alternative sgRNAs delivered as RNP complexes to primary human macrophages. Knockout rates were determined by sequencing (blue bars), and PrgI+PA-induced ASC specking rates were determined by anti-ASC antibody staining (red bars). Data from four biological replicates based on different human donors are shown. **P < 0.01; NS *P* > 0.05; two-sided *t*-test. NS, not significant.

Although monocytic cell-line-derived macrophages are a well-established platform to study cell-autonomous immune activation, primary blood-derived macrophages have the full biological potential of physiologically relevant immune activation and, therefore, would be an ideal cell system for innate immunity research. However, these cells are difficult to transduce, and no long-term reporter-expressing or Cas9-expressing cell lines can be established. To establish pooled optical screening in primary human macrophages, we first modified the CRISPR droplet sequencing (CROPseq) iT7 sgRNA-expressing lentiviral vector to co-express a Caspase-1–CARD (C1C)–EGFP fusion as a reporter for inflammasome activation^24^ and co-packaged it with VPX–VPR proteins (Fig. 4a). Transducing freshly differentiated M-CSF macrophages for a pooled optical screening procedure (Fig. 4b), we observed transduction efficiencies of 30% and homogeneous reporter activation upon inflammasome stimulation by FACS and microscopy (Fig. 4c,d and Supplementary Fig. 1f), which could be automatically quantified by the ratio of high-frequency-filtered EGFP signal to overall EGFP signal in individual cells (Fig. 4e). We next optimized knockout generation efficiency. First, we sorted transduced cells for EGFP expression using a large nozzle (see Protocols) and nucleofected SpCas9 protein to transiently form functional CRISPR complexes inside of cells. Targeted NGS confirmed high allelic editing rates of 25–35%, predicting at least 5% of cells bearing a bi-allelic frameshift mutation (Fig. 4f). Bringing all established techniques together, we performed an end-to-end pooled optical perturbation screen using a set of 28 sgRNAs targeting five hits from our previous THP1 screens (see above), including non-targeting controls. To ensure protein decay upon genetic knockout, cells were stimulated with PrgI+PA 5 d after nucleofection. Six cycles of NIS-Seq were used to correlate perturbed genes to inflammasome activation defects (Fig. 4g), confirming anthrax toxin receptor 2 as the entry receptor for PrgI+PA with effect size matching genome editing frequencies (Fig. 4h), whereas, unexpectedly, neither *NLRC4* nor *NAIP* perturbations increased the frequency of non-activated cells across sgRNAs. Arrayed validation using different sgRNAs delivered as ribonucleoprotein (RNPs) to primary human macrophages confirmed *ANTXR2*-dependent but *NLRC4*-independent inflammasome activation (Fig. 4i), alluding to an unknown activation mechanism in primary human macrophages different from that in THP1-derived model cells.

### Barcode identification in human skin tissue

**Fig. 5:**
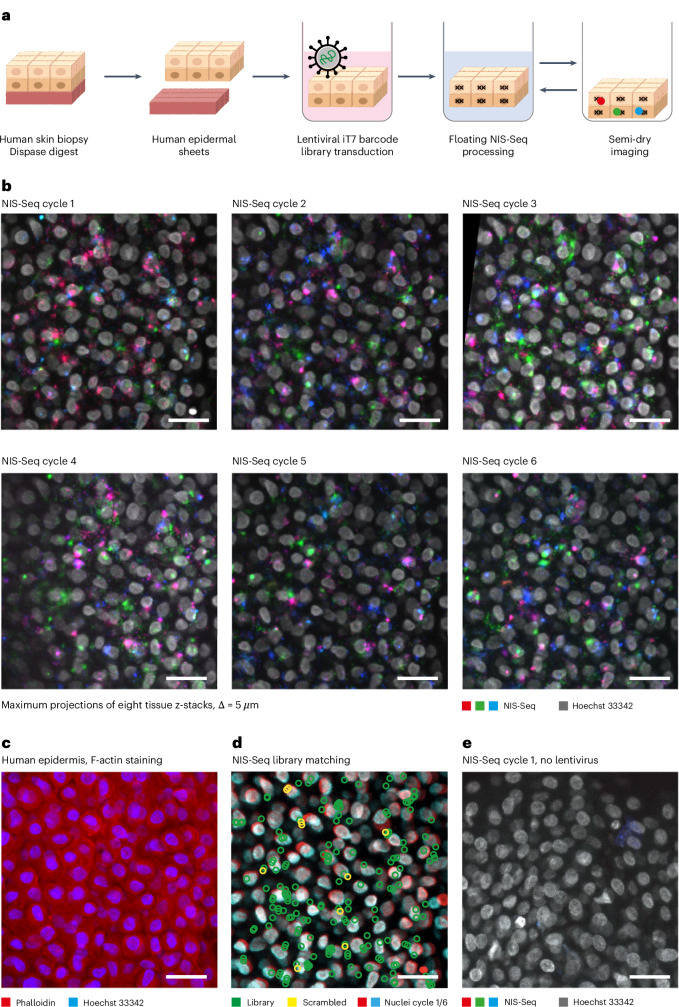
NIS-Seq optical barcode identification in human epidermal tissue sheets. **a**, Outline of preparing, transducing and sequencing human epidermal sheets using NIS-Seq. **b**, Six cycles of NIS-Seq barcode sequencing in human epidermal sheets transduced with a CROPseq-iT7 lentiviral library. Nuclear staining (gray) was performed at each cycle. Colors correspond to excitation lasers used to image NIS-Seq spots. Shown are maximum projections of eight tissue z-stacks starting at the bottom of the well distanced by 5 µm. Scale bar, 25 μm. Representative data from two experimental replicates are shown. **c**, Epidermal sheet shown in **b** stained for F-actin by phalloidin-AF670 (red). Scale bar, 25 μm. **d**, Epidermal sheet shown in **b** with indicated NIS-Seq signals matching members of the library used (green) or a scrambled library with the same base composition (yellow). Nuclei images from the first cycle (red) and sixth cycle (cyan) were overlayed in the background to visualize local tissue deformation artifacts. Instead of high-pass frequency filtering NIS-Seq data for sequence calling, images were low-pass filtered to compensate for imperfect alignments. Scale bar, 25 μm. **e**, One cycle of NIS-Seq barcode sequencing in human epidermal sheets as in **a** and **b** without prior lentiviral library transduction. Scale bar, 25 μm.

To test whether NIS-Seq can be used to identify barcodes in human tissue, we transduced epithelial sheets from human skin biopsies with the previously established lentiviral library encoding for 28 sgRNAs and C1C–EGFP (Fig. 5a). As live epidermal sheets are floating in media, we performed all subsequent fixation and NIS-Seq steps floating but removed the media for imaging eight z-planes starting from the bottom of the well. Images were rotated and translated to map cells across cycles (Fig. 5b) and F-actin staining (Fig. 5c), which confirmed specific mapping of NIS-Seq signals to the sgRNA library as compared to a scrambled control library but also revealed some local distortion artifacts due to the flexibility of the tissue (Fig. 5d). Non-transduced control tissue from the same donor did not yield NIS-Seq signals (Fig. 5e), confirming specific barcode detection in human tissue transduced with a lentiviral library.

### Zero-knowledge imaging data analysis

**Fig. 6:**
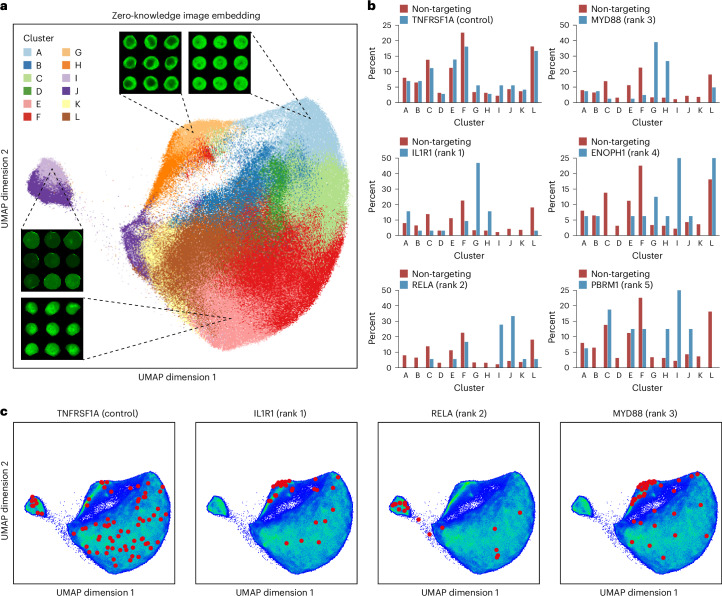
Zero-knowledge pooled optical screening analysis. **a**, p65–mNeonGreen channel images of 712,146 HeLa cells stimulated with IL-1β were embedded using the SwinV2-T computer vision model pre-trained on a general image dataset, *k*-means clustered based on 768 penultimate layer activations and visualized using UMAP dimensionality reduction. **b**, Cluster representation among top-ranking perturbed genes, identified by zero-knowledge image clustering. Perturbed genes covered by at least 15 cells were ranked by the maximum over representation of any cluster as compared to non-targeting control cells. **c**, Single-cell localizations of top-ranking outlier genes visualized on the same UMAP embedding as used in **a**.

The genetic screens performed using NIS-Seq relied on known protein translocation or aggregation events as phenotypic readouts. Interpreting screening data is largely based on human inspection of imaging data and optimization of a quantitative metric for functional perturbation of single cells, such as the Pearson correlation coefficient between nuclear DNA staining and fluorescent protein signals. To explore if we can supplement human intuition and knowledge with a machine learning model, we embedded a screening replicate comprising 712,146 mapped images of single HeLa cells stimulated with IL-1β using a modified SwinV2-T^25^ model, which was not trained on any labeled or unlabeled cellular images. For each cell, we extracted a 768-dimensional vector embedding from an internal layer of the model, based on which nearest neighbor cells revealed strong visual similarity across regions of a uniform manifold approximation and projection (UMAP) visualization (Fig. 6a). Clustering the perturbed cells in 768-dimensional space revealed heterogenous cluster representation across perturbed genes, of which the top three genes ranked by a metric of skewed cluster representation matched known pathway members (Fig. 6b). Of note, one of these (*RELA*) had been missed by rational analysis of the screening data before. Although perturbation of *RELA* did not affect the DNA-to-p65 correlation metric used in Fig. 2, zero-knowledge analysis mapped *RELA* perturbation to a strong downregulation of p65 reporter expression (cluster I, J). Visualizing cells without prior knowledge about the pathway studied or the reporter used provides an intuitive way to explore functionally perturbed cells (Fig. 6c) and may offer an alternative approach to the interpretation of large-scale optical screening datasets.

## Discussion

Although efficient editing of single genomic loci for functional genomics studies was possible with predecessor technologies of CRISPR, genome-scale perturbation screening has been revolutionized by the programmable nature of CRISPR nucleases through short lentiviral sgRNA expression cassettes. Perturbation screening enables systematic discovery of genetic determinants of biological processes^4–8^. NIS-Seq expands the applications of optical perturbation screening toward almost any nucleated cell. It is compatible with high cell density, high library complexity and highly dynamic phenotypes down to timescales of minutes. Thus, biological processes can be mapped to involved genes quantitatively and kinetically, not only identifying critical pathway members but also reading out genetic rheostats or pathway intersections. NIS-Seq is expected to be compatible with any phenotype observable by microscopy, including subcellular transport, cell migration, dynamic oscillation, protein complex formation, RNA splicing or single-molecule RNA localization^26^, optionally using newly developed super-resolution^27^ or non-optical microscopy techniques^28^.

We demonstrated the broad applicability of NIS-Seq by performing four genome-scale optical perturbation screens in cell lines, two in THP1-derived macrophages, which could not be screened using previous in situ sequencing protocols, and one small-scale targeted perturbation screen in primary human macrophages from the blood of healthy donors. The genome-scale screens comprised, on average, 45–88 cells per gene, which would be considered underpowered by previous reports; nevertheless, we identified the majority of known essential pathway members. To elucidate the optimal cell numbers required for future screens, repeated statistical re-analysis of downsampled screening data confirmed that screens approach saturation at approximately 100 cells per gene for detecting significant hits with an effect size of log_2_ > 1 and a similar phenotype distribution (Supplementary Fig. 1e). In contrast to enrichment-based CRISPR screening, optical pooled screens provide a single-cell distribution of phenotypes for each targeted gene as well as non-targeting controls, which may explain the high statistical power from sparse screening data even after FDR correction.

Typically, 40–60% of nuclei can be mapped to a reference library using NIS-Seq, which may be limited by several factors. First, cells transduced with a truncated lentiviral genome might express the resistance marker but lack the sgRNA cassette. Second, a fraction of library members is expected to be mutated due to PCR errors when cloning the library or polymerase errors during lentiviral packaging and transduction. Third, nuclei that display more than one bright spot are excluded from analysis, which may occur by integration of multiple viral genomes in a single cell. Lastly, NIS-Seq base-calling errors can limit the rate of perfect reference mapping.

To enable long-timeframe live imaging screens, an imaging sequence could be used acquiring low-magnification overview images in regular intervals between high-resolution imaging to keep track of cellular movements. NIS-Seq will enable not only loss-of-function screening but also CRISPR activation, cDNA overexpression or artificial intelligence (AI)-designed minibinder^29^ profiling, and help to identify optimized induced pluripotent stem cell (iPSC) differentiation protocols based on optical cell type identification^30^. As demonstrated here, NIS-Seq enables barcode identification even in primary human tissue, which will be useful for physiologically relevant genetic perturbation screens.

NIS-Seq requires 5 d of hands-on time for performing a genome-scale screen. To enable throughput and minimize human errors as well as reagent use, we successfully automated all repetitive steps using an off-the-shelf pipetting robot, for which we provide all programs on our website. To reduce acquisition times of NIS-Seq sequencing cycles, we successfully tested base calling by switching only the excitation laser when using a multiband emission filter. In the future, antibody-based base detection using the coolMPS technology might further increase signal intensity, reduce amplification steps and reduce phasing artifacts^31^. In parallel to NIS-Seq, two related methods were developed, which achieve optical barcode detection in cells either using a hybrid U6/T7 promoter^32^ or by detection of a combination of longer marker sequences co-expressed with the sgRNA^33^. Together, these methods will expand the applications of optical pooled screening toward highly relevant primary cell types and tissues.

NIS-Seq raw image analysis can be performed using our open-source online web applications (https://jsb-lab.bio/opticalscreening/), which do not require local software installation, server hardware or coding experience to analyze genome-scale optical perturbation screens.

## Methods

### CROPseq-iT7 backbone

CROPseq-Guide-Puro^8^, which was a gift from Christoph Bock (Addgene, 86708), was PCR amplified with primers CROPseq_iT7_fwd/rev using Q5 polymerase. PCR products were DpnI digested, 5′ phosphorylated and circularized by T4 ligation. Transformants were sequence verified using Tn5-mediated whole-plasmid tagmentation and MiSeq sequencing. The NIS-Seq-compatible CROPseq-iT7 backbone was deposited to Addgene (no. 211699).

### Library cloning

Library cloning was performed as previously described by Joung et al.^34^. In brief, the Human Brunello CRISPR knockout pooled library, a gift from David Root and John Doench (Addgene, 73178)^20^, was PCR amplified with gRNA_library_fwd/rev primers. The target vector CROPseq-iT7 was digested with the restriction enzyme Esp3I. Subsequently, the purified PCR product was cloned into the digested vector using Gibson assembly. The assembled reactions were pooled and purified via a Zymo DNA Clean & Concetrator-25 column. The purified library was electroporated in eight replicates into Endura electrocompetent cells (Lucigen), using 50–100 ng µl^−1^ DNA and 25 µl of cells in 0.1-cm Bio-Rad cuvettes at 1,800 V, 10 µF and 600 Ω. Cells were directly recovered in 975 µl of pre-warmed recovery medium and incubated for 1 h at 37 °C and 300-r.p.m. shaking. Then, each culture was transferred to 1 L of LB medium containing 100 µg ml^−1^ ampicillin and grown overnight at 37 °C and 230-r.p.m. shaking. DNA was purified using a PureLink HiPure Plasmid Maxiprep Kit. Library coverage was determined by Illumina NGS using primers MiSeq_gRNA_fwd_S0-S8, MiSeq_gRNA_rev for target amplification and Barcoding_fwd/rev_1-96 for a secondary barcoding PCR using NEBNext PCR polymerase. The NIS-Seq-compatible CROPseq-iT7 Brunello library was deposited to Addgene (no. 223064).

### Tissue culture

HeLa cells and immortalized murine macrophages (iMacs) were cultivated in DMEM GlutaMAX media supplemented with 10% FCS and 10 µg ml^−1^ ciprofloxacin in a 37 °C incubator with 5% CO_2_. THP1 cells, murine melanoma (B16-F1), human melanoma (MaMel65) and colon carcinoma (MC-38) cells were cultivated in RPMI GlutaMAX media supplemented with 10% FCS and 10 µg ml^−1^ ciprofloxacin in a 37 °C incubator with 5% CO_2_. Primary human monocytes were cultivated for differentiation in RPMI GlutaMAX media supplemented with 10% FCS, 10 µg ml^−1^ ciprofloxacin (Sigma-Aldrich) and 50 U ml^−1^ M-CSF (ImmunoTools). THP1 cells expressing ASC–GFP from an NF-κB-dependent promoter were purchased from InvivoGen (thp-ascgfp).

### Library transduction and quality control

In total, 1.5 × 10^7^ HEK293T cells were transfected in a 15-cm tissue culture dish using Lipofectamine 2000 (Invitrogen) with 14.1 µg of Brunello_iT7 plasmid library, 7.0 µg of lentiviral packaging plasmid pMD2.G (Addgene, 12259) and 10.6 µg of lentiviral packaging plasmid psPAX2 (Addgene, 12260). After 4–6 h of incubation, the medium was changed. Forty-eight hours later, virus-containing supernatant was filtered through a 0.45-µm filter (Merck Millipore), aliquoted and stored at −80 °C. For each of four screening replicates, 8 × 10^6^ HeLa–Cas9–p65–mNeonGreen cells^10^ were transduced with 1 ml of lentivirus library and 10 µg ml^−1^ polybrene (Merck Millipore), aiming for a multiplicity of infection (MOI) of 0.1–0.5. After 1 d, cells were selected and induced with 3 µg ml^−1^ puromycin and 1 µg ml^−1^ doxycycline (Cayman Chemicals). Cells were split 1:3 when reaching confluency. For each of two macrophage screening replicates, 1.6 × 10^7^ THP1–ASC–GFP–Cas9 CASP1/8^DKO^ cells were transduced with 2 ml of lentivirus library and 10 µg ml^−1^ polybrene. The next day, cells were selected with 3 µg ml^−1^ puromycin. After 4–7 d of selection and induction, 1 million cells were lysed in 100 µl of direct lysis buffer at 65 °C for 10 min and 95 °C for 15 min^19^. Genomically integrated guide sequences were amplified using NEBNext PCR polymerase (NEB) for 18 cycles and primers MiSeq_gRNA_fwd_S0-S8 and MiSeq_gRNA_rev (Supplementary Table 1) in two replicates per library. After secondary barcoding PCR, purification and NanoDrop-based quantification, libraries were sequenced on an Illumina NextSeq 2000 using a P2 100-cycle cassette. Library members were counted using the online application https://www.jsb-lab.bio/LibCounter.htm.

### NIS-Seq

For genome-scale NIS-Seq perturbation screens, 24-well glass-bottom plates (Greiner Bio-One) were coated with 0.1% poly-l-lysine (w/v in water; Sigma-Aldrich) for 30 min at room temperature and washed three times with PBS. For HeLa screens, cells were seeded at 5 d or 18 d of doxycycline induction. Per replicate, 4 × 10^5^ HeLa–Cas9–p65–mNeonGreen Brunello-iT7 library cells were seeded per well and incubated overnight. The next day, cells were stimulated for 45 min with 30 ng ml^−1^ human recombinant IL-1β (rcyec-hil1b; InvivoGen) or 30 ng ml^−1^ human recombinant TNF (rcyc-htnfa; InvivoGen), respectively. Live-cell nuclei were stained with 2 µM Hoechst 33342, and membranes were stained with 200 ng ml^−1^ CellMask Plasma Membrane Stain Deep Red (Thermo Fisher Scientific). For THP1 screens, THP1–ASC–GFP–Cas9 CASP1/8^KO^ Brunello-iT7 cells were pre-differentiated overnight with 100 ng ml^−1^ phorbol myristate acetate (PMA) (InvivoGen). The next day, cells were washed and detached, and 8 × 10^5^ cells were seeded per well in PLL-coated glass-bottom plates. The next day, cells were primed with 200 ng ml^−1^ lipopolysaccharide (LPS) (Sigma-Aldrich) for 3 h, pre-incubated with 50 µM Z-VAD (MedChemExpress) for 30 min and stimulated with 7.5 µg ml^−1^ nigericin (Cayman Chemicals) for 1 h. Cell membranes were stained with 200 ng ml^−1^CellMask Plasma Membrane Stain Deep Red (Thermo Fisher Scientific). All live-cell phenotype images were acquired in DMEM FluoroBrite with 10 mM HEPES and 2 µM Hoechst 33342 using a ×20 objective for HeLa cells and a ×10 objective with z-stacks for THP1 cells. After phenotyping, cells were fixed and permeabilized with a 3:1 mixture of methanol and acetic acid for 20 min. The fixation was washed out with 1× PBS to avoid dehydration of the cells. Cells were washed with nuclease-free water before adding 200 µl of T7 IVT mix per well (T7 MEGAscript 1× reaction mix; Thermo Fisher Scientific) for 3 h at 37 °C. After IVT, cells were fixed with 4% paraformaldehyde (PFA) in PBS for 20 min and washed with PBS-T (PBS + 0.1% Tween 20) two times. Reverse transcription and post-fixation steps were performed as described by Feldman et al. ^10^ using oRT_CROPseq_iT7 as reverse transcription primer. After post-fixation, cells were washed three times with PBS-T and incubated with a gap-fill Phusion mix (1× Ampligase buffer, 0.4 U µl^−1^ RNase H, 100 nM padlock probe oPD_CROPseq_iT7, 0.0125 U µl^−1^ NEB Phusion polymerase, 0.5 U µl^−1^ Ampligase, 0.05 mM dNTPs, 0.05 M KCl and 20% formamide) for 30 min at 37 °C and 45 min at 45 °C. Cells were then washed twice with PBS-T and incubated with an RCA mix overnight at 30 °C^10^. Cells were washed twice with PBS-T before hybridization of 1 µM of the in situ sequencing primer oSBS_CROPseq_iT7 in 2× SSC for 5 min at 37 °C. Cells were washed with MiSeq buffer PR2 (Illumina), and perturbation barcodes were analyzed by sequencing-by-synthesis using reagents from used Illumina NextSeq 2000 P2 cassettes. For each of 14 cycles, cells were incubated with the nucleotide incorporation mix for 3 min at 60 °C, followed by three rounds of five washes with PR2, each with 5-min incubation at 60 °C. The incorporated nucleotides were imaged after the addition of 200 ng ml^−1^ Hoechst 33342 in PR2. After each imaging cycle, fluorescent nucleotides were cleaved and de-blocked by incubation with NextSeq cleavage mix for 3 min at 60 °C, three washes with PR2, incubation for 2 min at 60 °C and three additional washes before the next cycle of incorporation. Robotic heating steps were performed at 50 °C instead of 60 °C. For a detailed step-by-step protocol, see Supplementary Protocol 1.

### Imaging

All images were acquired using a Nikon Ti2 body equipped with a Yokogawa CSU-W1 spinning disc unit connected to Lumencor CELESTA multimode lasers with wavelengths of 405 nm (nuclear staining), 477 nm (sequencing channel 1, mNeonGreen and GFP), 546 nm (sequencing channel 2) and 638 nm (sequencing channel 3 and CellMask deep red). Emission filters used included Chroma ET450/50 (nuclear staining), Chroma ET525/50 (sequencing channel 1, mNeonGreen and GFP), 572/28 BrightLine HC (sequencing channel 2) and 680/42 BrightLine HC (sequencing channel 3, CellMask deep red). Exposure times were 90 ms for all channels except p65–mNeonGreen, ASC–GFP and C1C–EGFP, which were exposed for 150 ms. Objectives used were a Nikon ×10 CFI P-Apo, a Nikon ×20 CFI P-Apo or a Nikon ×40 CFI Apo ×40 WI with or without a ×1.5 tube lens inserted into the light path. A Hamamatsu Orca Flash4.0 LT+ camera was used in electronic shutter mode at full resolution (2,048 × 2,048). For a detailed step-by-step protocol, see Supplementary Protocol 2.

### Image analysis of NIS-Seq data

Raw images of up to 14 NIS-Seq cycles were aligned by FFT-accelerated cross-correlation of nuclear staining images. Spots were detected by summing up all sequencing channels across the first three sequencing cycles, high-pass filtering, local maximum detection and brightness thresholding. Spot sequence information was aggregated across 5 × 5 pixels for every spot after high-pass filtering and eliminating negative values. Channel unmixing was performed by multiplying the channel vector of each cycle with the inverse matrix of average base-wise channel intensities. Non-G bases were called by the maximum of unmixed channels, whereas Gs were called at cycles with all unmixed intensities below 20% of the spot’s maximum unmixed intensity across all cycles. Sequences were assigned to the dictionary of known sequences (Brunello sgRNA sequences reverse complemented), allowing zero or one mismatch and no ambiguities. Dictionary-matched and dictionary-corrected spot sequences were assigned to nuclei, whose outlines were defined by Cellpose using the ‘nuclei’ model^35^, requiring the dominant sequence to make up more than two-thirds of total intensity of library spots in a given nucleus and the maximum signal intensity per nucleus across channels and sequencing cycles to pass a numeric threshold (7 × 10^5^ for all cell types except 2 × 10^5^ for iMacs). For a detailed step-by-step protocol, see Supplementary Protocol 3.

### Image analysis of live phenotyping data

z-stacks were collapsed by averaging where applicable. Cell and nuclear outlines were defined by CellPose using the ‘cyto2’ and ‘nuclei’ models^35^. Nuclear translocation of mNeonGreen was quantified by calculating the Pearson correlation between nuclear staining and mNeonGreen fluorescence across pixels pertaining to each cell. GFP specking was quantified by local background subtraction (see below), high-pass filtering of fluorescent images, eliminating negative valued pixels and calculating the mean fluorescence across pixels pertaining to each cell before and after high-pass filtering. Cells with low mNeonGreen or GFP expression were excluded from downstream analysis. For local background subtraction, images were downsampled 8 × 8-fold. For each pixel in the full-resolution image, the local minimum across the 9 × 9 closest pixels in the downsampled image was subtracted, after which negative values were set to zero.

### Analysis of HeLa genome-scale NIS-Seq perturbation screening data

Pairs of live phenotyping and NIS-Seq nuclear images acquired at corresponding stage positions were fine-mapped using FFT-accelerated cross-correlation. Phenotyping nuclei were assigned to the closest nucleus in shifted NIS-Seq data by centers of gravity, with a maximum movement distance of 11.1 µm and with the nuclear area matching within a two-fold margin. Any ambiguously mapping nuclei were excluded from further analysis.

### Analysis of THP1 genome-scale NIS-Seq perturbation screening data

THP1 phenotypes were imaged using a ×10 objective with z-stacking to better cover small ASC specks across the cellular cytosol. Furthermore, live nuclear imaging turned out to be affected by the stimulation of cells. Therefore, the assignment of phenotype and NIS-Seq images described above for HeLa cells was modified. Instead of using live nuclear images, cell outlines derived using CellPose from z-aggregated membrane staining were shrunk by 5 pixels to predict the location of the nuclei. Potential pairs of phenotyping and NIS-Seq imaging fields of view were identified based on microscope stage positions. Images were scaled according to the relative magnification used and coarsely mapped at 8 × 8-downsampled resolution using FFT-accelerated cross-correlation. Best-correlated pairs of fields of view were fine-mapped at 2 × 2-downsampled resolution using FFT-accelerated cross-correlation. All subsequent analysis steps were performed as described above for HeLa cells.

### Generation of single-gene perturbation cell lines for validation assays

sgRNAs were selected from the Toronto human knockout pooled library (TKOv3) and ordered as DNA oligonucleotides (Integrated DNA Technologies (IDT); Supplementary Table 1). Oligonucleotides were inserted into the CROPseq-iT7 lentiviral backbone using Golden Gate cloning. Purified and sequence-verified plasmids were used to produce lentivirus in HEK293T cells. Transduced cells were selected with puromycin for 3–5 d and screened for the phenotype of interest.

### Primary human macrophage generation

Leukocyte-enriched buffy coats were mixed 1:1 with DPBS within 8 h after donation. Then, 15 ml of Ficoll-Paque Plus (Cytiva) was overlayed with 35 ml of blood–PBS mixture and centrifuged at 700 g for 20 min at 25 °C with disabled brakes. The upper serum-containing layer was removed, and the mononuclear cell layer was transferred to a fresh 50-ml reaction tube, filled up to 50 ml with DPBS and centrifuged at 340 g for 10 min at 25 °C. The supernatant was discarded, and the cells were resuspended in 50 ml of DPBS and pelleted for 5 min. Cells were resuspended in 500 µl of MACS buffer (DPBS, 2% FCS, 1 mM EDTA) and mixed with 100 µl of CD14 MicroBeads (Miltenyi). After incubation for 15 min at 4 °C, 25 ml of MACS buffer was added, and the cells were centrifuged at 340 g for 5 min. To separate CD14^+^ monocytes from the cell population, the cell pellet was gently resuspended in 3 ml of MACS buffer and added onto a LS column (Miltenyi Biotec) on a QuadroMACS Separator (Miltenyi Biotec). The column was washed three times with 3 ml of MACS buffer before eluting the cells in 3 ml of MACS buffer. After centrifuging at 340 g for 5 min, cells were recovered in 10 ml of complete media (RPMI GlutaMAX, 10% FCS, 1% sodium pyruvate, 1% penicillin–streptomycin) and counted. In total, 10^7^ cells were incubated in 5 ml of complete media in the presence of 50 U ml^−1^ recombinant human macrophage colony-stimulating factor (rhM-CSF) (ImmunoTools) in six-well plates (Thermo Fisher Scientific) for 4 d.

### Primary human macrophage harvesting

Differentiated macrophages were harvested by collecting the cells in suspension in a 50-ml reaction tube. Partially adherend cells were detached by adding 1 ml of DPBS containing 5 µM EDTA and incubation for 5 min at 37 °C. Both fractions were collected in a 50-ml reaction tube, counted and centrifuged at 340*g* for 5 min. In total, 2.5 × 10^6^ cells were seeded in 2 ml of complete media containing 50 U ml^−1^ rhM-CSF in six-well plates (Greiner Bio-One) and incubated for 24 h.

### Lentiviral production for primary human macrophage transduction

One day before transfection, 15 × 10^6^ HEK293T cells were seeded in 30 ml of complete media (DMEM GlutaMAX, 10% FCS, 1% penicillin–streptomycin) in a 15-cm culture dish. Directly before transfection, the media were changed to 20 ml of 2% FCS-containing media. For transfection, 120 µl of Lipofectamine 2000 was incubated with 1,660 µl of Opti-MEM for 5 min and mixed with the following plasmids in a total volume of 1,780 µl of Opti-MEM: 7,040 ng of pCAGGS-VPx-VPr, 7,040 ng of lentiviral cargo plasmid, 10,560 ng of psPAX2 and 7,040 ng of pMD2.G. After 20 min, the transfection mix was added dropwise to the cells. The media were replaced after 4–6 h with complete media, and cells were incubated for 48 h at 37 °C and 5% CO_2_. The supernatant was filtered through a 0.45-µm syringe filter (VWR) and mixed with 1/3 volume of Lenti-X Concentrator (Takara). The mixture was incubated on ice for 45 min and centrifuged at 1,500 g for 45 min. The pellet was resuspended in 1/6 of the initial volume.

### Primary human macrophage transduction

One day after harvest, the culture volume was reduced to 1 ml, and the macrophages were transduced with 400 µl of concentrated lentivirus in the presence of 10 µg ml^−1^ polybrene (Millipore). The media were changed after 6 h to 2 ml of complete media.

### Sorting of EGFP^+^ primary human macrophages

Cells were harvested and adjusted to a concentration of 4 × 10^6^ cells per ml^−1^ in complete media containing 20% FCS and 50 U ml^−1^ rhM-CSF. Cells were sorted using a Sony MA900 with a 130-µm sorting chip in sorting mode ‘yield’ into media containing 50% FCS and 50 U ml^−1^ rhM-CSF. Cells were pelleted, resuspended in complete media and seeded at a maximum density of 2 × 10^6^ cells per milliliter in six-well plates.

### Cas9 nucleofection of primary human macrophages

Harvested cells were washed in DPBS, counted and pelleted. In total, 2.5 × 10^6^ cells were resuspended in 20 µl of P3 Primary Cell Nucleofector Solution (Lonza). Cells were nucleofected with 50 µg of SpCas9 v3 (IDT) in a 20-µl Nucleocuvette Strip (Lonza) using program CM-137. Cells were incubated in the strip for 5 min at 37 °C and subsequently recovered in warm complete media (50 U ml^−1^ rhM-CSF, no antibiotics). Cells were seeded into µ-Plate 96-squared-well plates (ibidi) at a density of 0.75 × 10^5^ cells per milliliter. One day after nucleofection, the media were changed to complete media (RPMI GlutaMAX, 10% FCS, 1% sodium pyruvate, 1% penicillin–streptomycin, 50 U ml^−1^ rhM-CSF).

### Inflammasome stimulation in primary human macrophages

Infected and nucleofected macrophages were primed with 20 ng ml^−1^ LPS (InvivoGen) for 3 h. To inhibit caspase-mediated cell death pathways, cells were incubated with 40 µM VX-765 (InvivoGen) and 50 µM Z-VAD-FMK (MCE, HY-16658B) for 30 min before stimulation. To activate an inflammasome, 1 µg ml^−1^ PA (BIOZOL, LBL-171E) together with 10 ng ml^−1^ of LFn-PrgI were added to the cells. After 1.5 h, cells were imaged in 75 µl of pre-warmed FluoroBrite DMEM (Gibco) in the presence of 1× CellMask DeepRed plasma membrane stain (Invitrogen) and 1 µg ml^−1^ Hoechst 33342. After imaging three z-stacks at a distance of 2.5 µm, cells were fixed using MeAA.

### Preparation, transduction and sequencing of human epidermal sheets

Leftover skin tissue from clinical procedures was cut using a 6-mm biopsy punch (Kai Medical). Samples were incubated in 5 U ml^−1^ Dispase (Gibco, sterile filtered) for 2 h at 37 °C and washed once in PBS. Epidermal sheets were separated by gently pulling apart dermis and epidermis using sterile metal tissue tweezers. Epidermal sheets were kept in ex vivo culture media (DMEM GlutaMAX, 10% FCS, 1% penicillin–streptomycin, 25 µg ml^−1^ gentamycin, 1% amphotericin B) and infected with a concentrated CROPseq-iT7 lentiviral library (see above) in the presence of 10 µg ml^−1^ polybrene for 20 h at 37 °C. Epidermal sheets were washed twice in PBS and fixed in 4% PFA for 45 min at room temperature. Subsequently, sheets were washed three times in PBS, de-crosslinked in 0.5 M NaCl for 2 h at 65 °C and permeabilized in 70% ethanol for 30 min at room temperature. NIS-Seq was performed as described above with sheets floating in-solution in a 96-well plate (ibidi); imaging was performed by removing the liquid and centering the tissue in the well. Nuclei were stained after each cycle with 2 μM Hoechst 33342. F-actin was stained using 0.2 µM phalloidin-AF670.

### Zero-knowledge image analysis of screening image data

To identify perturbations that lead to visual cellular changes, we performed zero-knowledge image analysis using deep learning. We used the established computer vision model SwinV2-T^25^, which was pre-trained on the ImageNet-1K dataset, modified it to embed monochromatic p65–mNeonGreen channel images of perturbed cells and removed the classification head. For each cell, we extracted the penultimate layer activations of the model, resulting in 768-dimensional vector embeddings. Using the *k*-means algorithm, the cells were divided into 200 clusters. Agglomerative clustering was applied to the centroids of those clusters to obtain 12 clusters. For illustration and visual inspection purposes, the 768-dimensional space of embeddings was projected to an auxiliary two-dimensional space using the UMAP algorithm.

### Ethics statement

Buffy coats from healthy donors were obtained according to protocols accepted by the institutional review board at the University Hospital Bonn (local ethics vote Lfd. Nr. 075/14). Human skin biopsies were obtained from leftover skin of individuals undergoing plastic surgery at the University Hospital Bonn with informed consent of patients and according to protocols accepted by the institutional review board at the University Hospital Bonn (local ethics votes Lfd. Nr. 037/06, including amendments 2 and 3).

### Reporting summary

Further information on research design is available in the Nature Portfolio Reporting Summary linked to this article.

## Online content

Any methods, additional references, Nature Portfolio reporting summaries, source data, extended data, supplementary information, acknowledgements, peer review information; details of author contributions and competing interests; and statements of data and code availability are available at 10.1038/s41587-024-02516-5.

## Supplementary information

Supplementary Information Supplementary Fig. 1 and Supplementary Protocols 1–3.

Reporting Summary

Supplementary Tables 1 and 2 Oligonucleotides and primers used in this study. Overview of screening imaging datasets.

Supplementary Code 1 Source code of NIS-Seq image analysis and Python scripts used in Figs. 1e,f, 2a,d and 3a,e.

## Data Availability

NIS-Seq screening data are available through Zenodo^36^. Example raw imaging data are available at https://jsb-lab.bio/opticalscreening/. Raw imaging data (details in Supplementary Table 2; >1 TB) are available upon reasonable request.

## Code availability

A web-based NIS-Seq image analysis application and source code are available at https://www.jsb-lab.bio/opticalscreening/. Python notebooks used to create Figs. 1e,f, 2a,d and 3a,e and the source code of the web-based applications are available at GitHub^37^ as well as in the supplementary material. The ELISA plate reader was operated using the latest version of BioTek Gen5 software. The Illumina MiSeq and Illumina NextSeq 2000 sequencers were operated by the current version of the respective Illumina control software. The spinning disc microscope was operated using custom-developed software that is available at https://jsb-lab.bio/jsMicroscope/.
