# Supplementary material for: NIS-Seq enables cell-type-agnostic optical perturbation screening
Source: Nat Biotechnol. 2024 Dec 19;43(8):1337–47. doi: 10.1038/s41587-024-02516-5 (PMC12339361; doi:10.1038/s41587-024-02516-5)
Supplement: Supplementary file 1 — Supplementary Fig. 1 and Supplementary Protocols 1–3. [file 41587_2024_2516_MOESM1_ESM.pdf]

nature biotechnology

Supplementary information https://doi.org/10.1038/s41587-024-02516-5

NIS-Seq enables cell-type-agnostic optical perturbation screening

In the format provided by the authors and unedited

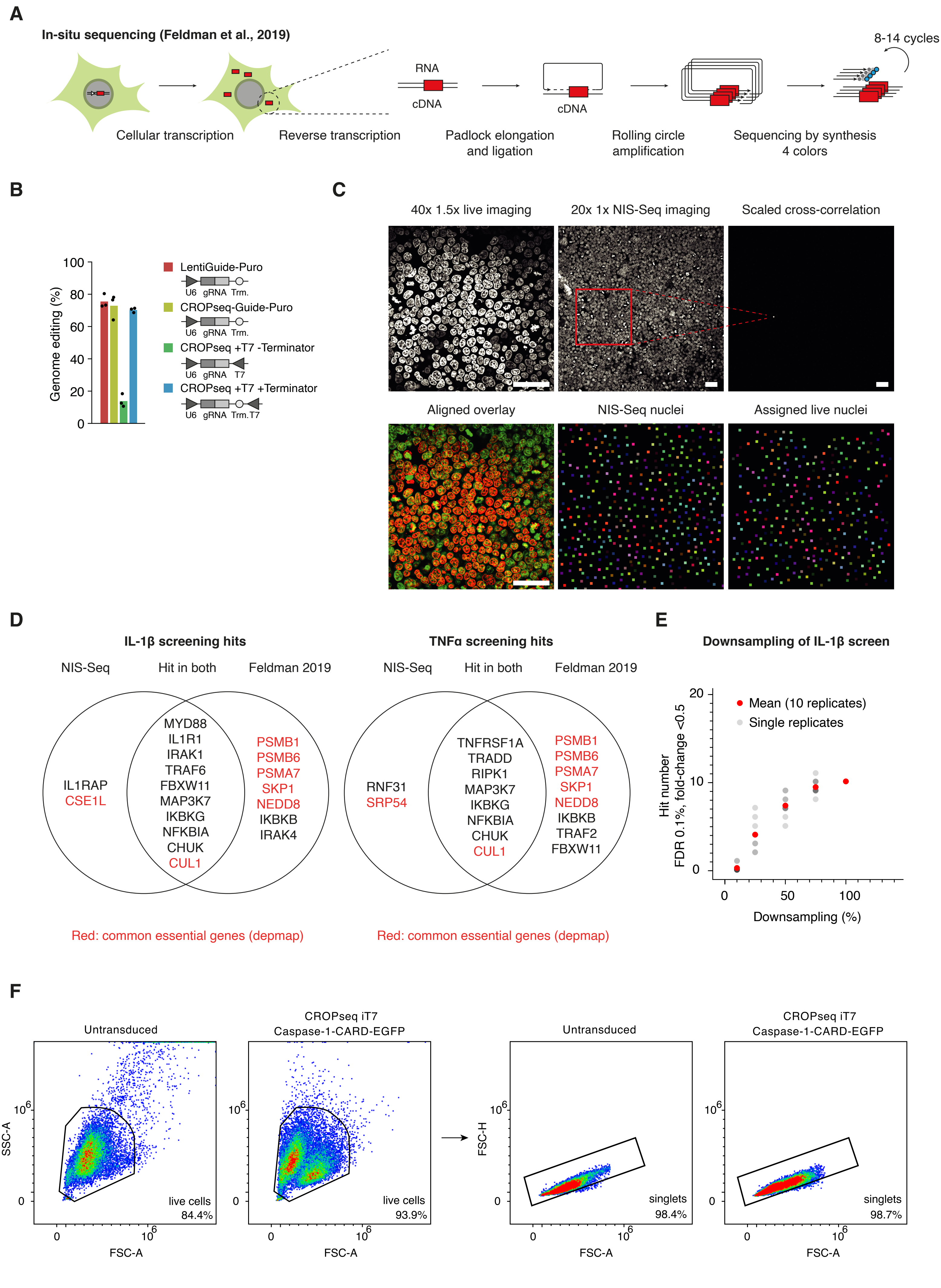

SUPPLEMENTARY FIGURE 1

Supplementary Figure 1 | (A) Outline of established in-situ sequencing reaction steps relying on barcoded mRNA [10], as compared to NIS-Seq outlined in Fig. 1. **(B)** Genome editing efficiencies of widely-used sgRNA-expressing lentivirus designs compared to constructs with an additional T7 promoter inserted for NIS-Seq in reverse-orientation. Genome editing efficiencies at three independent loci were assessed by NGS in HeLa-Cas9 cells transduced with indicated lentiviral constructs after four days of Puromycin selection and Cas9 induction. **(C)** Nuclei assignment between live cell imaging and NIS-Seq data across different objectives and timepoints. Nuclear staining images from HEK 293T cells are mapped by two-dimensional FFT-accelerated high-pass filtered cross-correlation (top right panel). Overlay of nuclear signal reveals slight dislocation of nuclei between imaging timepoints (bottom left; red image, nuclei at approx. 30 minutes before fixation; green image, post-fixation nuclei). Centers of gravity of CellPose-defined nuclei are assigned to nearest neighbors and ambiguous assignments are removed (bottom center and right). Assigned nuclei are color-coded with the same random color. Scale bar, 50 µm. Representative data from two experimental replicates are shown. **(D)** Precision and recall calculation of NIS-Seq HeLa screening results assuming a similar screen by Feldman et al.[10] as ground truth. Considering only genes covered in both screens and not listed as common essential gene in DepMap.org, the average precision of NIS-Seq screening hits is 100% (9/9 and 7/7 hits), and the average recall is 75.9% (9/11 and 7/10 hits). **(E)** Estimation of cell number saturation in the exemplary genome-wide NIS-Seq screen shown in Fig. 2A. Screening data were randomly down-sampled in ten replicates to relative cell numbers indicated on the x-axis. Based on the same statistical analysis as used in Fig. 2A, the number of significant screening hits with a mean fold-change of less than 0.5 at a false-discovery rate of 0.1% were determined for each sampling. Shown are single results as well as the mean hits numbers across ten replicate samplings. **(F)** Exemplary gating strategy of FACS data shown in in Fig. 4. Live cells were gated in FSC-A / SSC-A representation. Subsequently, singlets were gated in FSC-A/FSC-H representation.

Supplementary Protocol 1 I Lab Protocol for NIS-Seq Optical Pooled Screens v1.0 I 8/24

Caroline I. Fandrey and Marius Jentzsch, Schmid-Burgk Lab, University of Bonn
http://jsb-lab.bio/opticalscreening/

Biological Materials

- HeLa cell line (ATCC, #CCL-2), transduced with pR14_p65-mNeonGreen (Addgene Plasmid, #127172) - kindly provided by the Blainey Lab and the Cheeseman Lab
- THP1 ASC-GFP (Invivogen, #thp-ascgfp), transduced with lentiCas9-Blast (Addgene Lentiviral Prep, #52962-LV)
- NEB 5-alpha Competent E. coli (NEB, #C2987H)
- Endura Electrocompetent Cells (Biosearch Technologies, #60242-2)

Reagents

Custom sgRNA libraries and cloning

- CROPSeq T7 Brunello sgRNA library (Addgene, deposition in progress)
- sgRNA lentiviral delivery vector: CROPseq-iT7 (Addgene Plasmid, #211699)
- sgRNA oligos for cloning into delivery vector (IDT, see Supplementary Table 1)
- PCR primer pairs for library amplification (IDT, see Supplementary Table 1)
- NEBNext High-Fidelity 2X PCR Master Mix (NEB, #M0541L)
- FastDigest Esp3I (Thermo Fisher Scientific, #FD0454)
- StickTogether DNA Ligase Buffer (NEB, #B0535S)
- DTT (Thermo Fisher Scientific, #P2325)
- BSA (NEB, #B9200)
- T7 DNA Ligase (NEB, M0318L)
- E-Gel EX 2% (Thermo Fisher Scientific, #G401002)
- GeneRuler 1 kb Plus DNA Ladder (Thermo Fisher Scientific, #SM1333)
- QIAquick PCR Purification Kit (Qiagen, #28106)
- QIAprep Spin Miniprep Kit (Qiagen, #27106)
- Zymoclean Gel DNA Recovery Kit (Zymo Research, #D4007)
- Purelink HiPure Plasmid Maxiprep Kit (Thermo Fisher Scientific, #K210007)
- UltraPure DNase/RNase-Free Distilled Water (Thermo Fisher Scientific, #10977035)

NGS

- NGS validation primers and indexing primers (IDT, see Supplementary Table 1)
- Miseq Reagent Nano Kit, v2, 300 cycles (Illumina, #MS-103-1001)
- PhiX Control Kit v3 (Illumina, #FC-110-3001)
- Sodium Hydroxide solution, 1 N (AppliChem, #182415.1211)

Mammalian cell culture

- Dulbecco's Modified Eagle Medium (DMEM), high glucose, GlutaMAX Supplement (Thermo Fisher Scientific, #61965-059)
- RPMI 1640 Medium, GlutaMAX Supplement (Thermo Fisher Scientific, #61870-044)
- Dulbecco's PBS (Thermo Fisher Scientific, #14190250)
- Ciprofloxacin HCl (Sigma-Aldrich, #PHR1044-1G)
- Fetal Bovine Serum (FBS) (SERANA, Lot No.: 45030122FBS)
- Phorbol Myristate Acetate (Invivogen, #tlrl-pma)
- Trypan Blue Solution 0.4% (Sigma-Aldrich, #93595-50ML)
- Sodium Pyruvate 100mM (Thermo Fisher Scientific, #11360-039)
- Penicillin Streptomycin (Thermo Fisher Scientific, #15140122)
- Trypsin-EDTA (0,25%) (Thermo Fisher Scientific, #25200072)

Cas9 Cell Line Creation

- Blasticidin (Invivogen, #ant-bl-05)
- lentiCas9-Blast (Addgene Lentiviral Prep, #52962-LV)

Lentivirus Production and Transduction

- pMD2.G (Addgene, #12259)
- psPAX2 (Addgene, #12260)
- VPX-VPR (created by Florian I. Schmidt)
- Opti-MEM I Reduced Serum Medium (Thermo Fisher Scientific, #31985062)
- Lipofectamine 2000 Transfection Reagent (Thermo Fisher Scientific, #11668019)
- Polybrene (Millipore, #TR-1003-G)
- Puromycin dihydrochloride (Thermo Fisher Scientific, #A1113803)

Primary human monocyte isolation

- Ficoll Paque Plus (Cytiva, #17-1440-02)
- EDTA 0.5M, pH 8.0 (Thermo Fisher Scientific, #AM9260G)
- MACS CD14 Microbeads (Miltenyi Biotec, #130-050-201)
- LS Columns MACS Cell Separation (Miltenyi Biotec, #130-042-401)
- 30 um Pre-Seperation Filter (Miltenyi Biotec, #130-041-407)
- MACS MultiStand (Miltenyi Biotec, #130-042-303)
- QuadroMACS Starting Kit (LS) (Miltenyi Biotec, #130-091-051)
- Recombinant Human M-CSF (PeproTech, #300-25)
- Nunclon Delta 6 Well Plate (Thermo Fisher Scientific, #140685)

Primary human monocyte nucleofection

- P3 Primary Cell 4D-Nucleofector X Kit S (Lonza, #V4XP-3032)
- Alt-R S.p. Cas9 Nuclease V3 (IDT, #1081059)

Live-cell phenotyping

- pR14_p65-mNeonGreen (Addgene Plasmid, #127172)
- FluoroBrite DMEM (Thermo Fisher Scientific, #A1896701)
- Hoechst 33342 Solution (Thermo Fisher Scientific, #62249)

- CellMask Deep Red Plasma Membrane Stain (Thermo Fisher Scientific, #C10046)
- Recombinant human TNF-a (Invivogen, rcyc-htnfa)
- Recombinant human IL-1ß (Invivogen, rcyec-hil1b)
- Nigericin (sodium salt) (Cayman, 11437-10mg)
- Ethanol 100% (Carl Roth, #5054.4)
- LPS (InvivoGen, #tlrl-eblps)
- Protective Antigen "PA" (Biozol, LBL-171E)
- LFn-PrgI (produced by Eicke Latz, Florian Schmidt, Matthias Geyer)
- Z-VAD(OMe)-FMK (Biotrend, #HY-16658-5mg)
- VX-765 (Invivogen, #inh-vx765i-1)
- Methanol, anhydrous (Sigma-Aldrich, #322415-1L)
- Acetic Acid, (Sigma-Aldrich, #A6283-500ML)

Fixed Cell Phenotyping

- 16 % Formaldehyd (w/v), methanol-free (Thermo Fisher Scientific, #28908)
- Sodium Bicarbonate (Sigma-Aldrich, #S6014)
- DAPI (Sigma-Aldrich, #D9542-10MG)

NIS

- Dulbecco's PBS (Thermo Fisher Scientific, #14190250)
- UltraPure DNase/RNase-Free Distilled Water (Thermo Fisher Scientific, #10977035)
- 16 % Formaldehyd (w/v), methanol-free (Thermo Fisher Scientific, #28908)
- Ethanol 100% (Carl Roth, #5054.4)
- Triton™ X-100, 98 % (Thermo Fisher Scientific, #327371000)
- Tween 20 (Sigma-Aldrich, #P9416-100ML)
- Sodium Chloride (Sigma-Aldrich, #S3014)
- MEGAscript T7 Transkriptionskit (Thermo Fisher Scientific, #AM1333)
- RevertAid H Minus Reverse Transcriptase (Thermo Fisher Scientific, #EP0452)
- Deoxynucleotide (dNTP) Solution Mix (NEB, #N0447L)
- BSA (NEB, #B9200)
- Reverse Transcription Primer (IDT, see Sup. Tab. XY)
- RiboLock RNase Inhibitor (Thermo Fisher Scientific, #EO0384)
- 25% Glutaraldehyde (Sigma-Aldrich, G5882-10X1ML)
- Padlock Oligo (IDT, see Sup. Tab. XY)
- Formamide (Sigma-Aldrich, #F9037-100ML)
- Potassium Chloride (Sigma-Aldrich, #P9541-500G) – double-check
- RNAse H (NEB, #M0297L)
- Phusion High-Fidelity DNA Polymerase (NEB, #M0530L)
- Ampligase DNA Ligase (Biosearch Technologies, #A3210K)
- Glycerol (Sigma-Aldrich, #G5516-500ML)
- phi29 DNA Polymerase (Thermo Fisher Scientific, #EP0094)
- SBS Sequencing Primer (IDT, see Sup. Tab. XY)
- RNase-free 20× SSC buffer (Ambion, #AM9763)
- Hoechst 33342 Solution (Thermo Fisher Scientific, #62249)

- NextSeq 1000/2000 P2 Reagent 2 and Reagent 4 (not XLEAP) (Illumina, #20046811) – **CAUTION**: The protocol and microscopy filter sets are designed and optimized for the two-color based sequencing chemistry of Illumina's NextSeq1000/2000 platform. NextSeq cartridges are required to pass the on-board pre-run checks before extracting Reagent 2 and Reagent 4.
- MiSeq Reagent Nano Kit v2 PR2 buffer (Illumina, #MS-103-1003)

Consumables

- Eppendorf DNA LoBind Microcentrifuge Tubes, 1.5 mL (Eppendorf, #0030108051)
- Falcon Polypropylene Centrifuge Tubes 50 mL (Corning, #352070)
- Falcon Polypropylene Centrifuge Tubes 15 mL (Corning, #352096)
- PCR SingleCap 8er-SoftStrips 0.2 mL (Biozym, #710970)
- 96 PCR Plate half-skirted (Sarstedt, #72.1979.102)
- Opti-Seal Optical Sealing Foil (BIOplastics, #157300L)
- AlumaSeal (Excel Scientific, #F-96-100)
- Filtered sterile pipette tips (Rainin)
- Gene Pulser Electroporation Cuvettes 0.1 cm (Bio-Rad, #1652089)
- T75 Tissue Culture Flask (Sarstedt, #83.3911)
- 15 cm Cellstar TC Dish (Greiner, #639160)
- Serological Pipettes 25 mL (Sarstedt, #86.1685.001)
- Serological Pipettes 10 mL (Sarstedt, #86.1254.001)
- Serological Pipettes 5 mL (Sarstedt, #86.1253.001)
- 0.45 Syringe Filter (VWR, #76479-012)
- Sterican MIX Blunt Needle 1.2x40mm (Braun, #4038088-01)
- Syringe Omnifex LuerLock 50mL (Braun, #4917509F)
- CellStar 6-well TC Plate (Greiner, #657160)
- Glass-bottom SensoPlate 24-well Plate (Greiner, #662896)
- μ-Plate 96-well Square Microscopy Plate (Ibidi, #89626)
- CellCarrier 96-well Microscopy Plate (PerkinElmer, #6055302)
- Lense-Cleaning Tissues (Kimtech, #7558)
- Ampicillin (Carl Roth, #K029.4)
- LB-Agar (Lennox) (Carl Roth, #X965.3)
- Petri Dishes (Sarstedt, #82.1473.001)
- Biomek Tips 1025 uL, sterile filtered (Beckman Coulter, #B85955)
- SureFlow 300 mL Reagent Reservoir (Integra, #6309)

Equipment

- Eppendorf ThermoMixer C (Eppendorf, #5382000015)
- Eppendorf SmartBlock Plates (Eppendorf, #5363000039)
- Eppendorf ThermoTop (Eppendorf, #5308000003)
- Gene Pulser Xcell Microbial System (Bio-Rad, #1652662)
- Lonza 4D-Nucleofector X Unit (Lonza, #AAF-1003X)

- CellDrop FLi Automated Cell Counter (DeNovix, #31CD-FLI-UNLTD-W)
- Thermocycler 96-well Bio-Rad T100 (Bio-Rad, #1861096)
- Table-Top Microcentrifuges (Eppendorf, #5920R & #5804)
- E-Gel electrophoresis device (Invitrogen, #G8100)
- Gel Imaging System (Vilber Fusion FX7, #151113001)
- NanoDrop OneC (Thermo Fisher Scientific, #ND-ONEC-W)
- MiSeq System (Illumina)
- NextSeq 2000 (Illumina)
- Bacteria Incubator (Memmert, #in55)
- Orbital Shaker (Eppendorf, #2230000050)
- CO2 Incubator (PHCBI, #MCO-170AICUVH-PE)
- Biomek i7 Hybrid (MC + Span-8) with Enclosure (Beckman Coulter, #B87585)
- Inheco Static Peltier (Inheco, #A93938)
- Fiji, an image processing package of ImageJ, available via https://imagej.net/Fiji
- Epifluorescent Microscope Ti2-E (Nikon, #MEA54000) with motorized stage (#MEC56120)
- Nikon CFI P-Apo 10x Lambda (#MRD00105)
- Nikon CFI P-Apo 20X Lambda (#MRD00205)
- Nikon CFI Apo 40x WI Lambda-S (#MRD77400)
- OrcaFlash 4.0 Camera (Hamamatsu, #C13440-20CU)
- Yokogawa W1 SpinningDisk Unit (Yokogawa, CSU-W1-T1)
- Homogenizer VisiScope for Confocal CSU-W1 linkage to Ti2-E (Visitron Systems, #VS-HOM1000)
- Lumencor CELESTA Quattro nIR (#90-10696, Lasers: 405/477/546/638/749, Penta Line Splitter: 421/491/567/659/776, Penta Line Filter: 421/491/567/659/776)
- Suggested Emission Filters: Chroma ET450/50 (nuclear staining), Chroma ET525/50 (sequencing channel 1, mNeonGreen, GFP), 572/28 BrightLine HC (sequencing channel 2), 680/42 BrightLine HC (sequencing channel 3, CellMask deep red)
- Suggested Computer Hardware: >16GB RAM

NIS-Seq Workflow

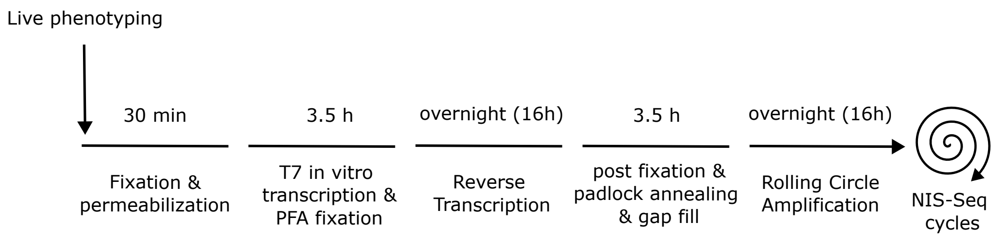

NIS-Seq Summary

After phenotyping the screening cells, a T7 polymerase-based in vitro transcription is performed. The inverted T7 promoter downstream of the sgRNA barcode sequence serves as a starting point for transcription and leads to amplification of the sgRNA coding sequence directly from the genomic DNA in the nucleus. The resulting IVT product is fixed in the cell nucleus and transcribed into cDNA by a reverse transcription reaction overnight. The subsequent post-fixation step ensures that the RT product also remains in the cell nucleus. The padlock oligo is then attached to the RT product and the gap between the padlock ends is filled with dNTPs. The resulting product serves as a template for the final step of rolling circle amplification overnight. Finally, the primer for in situ sequencing is annealed to the amplified DNA template, and the cell-specific barcode sequence can be analyzed over 14 sequencing cycles.

Step-by-step protocol NIS-Seq (96-well ibidi or 24-well Greiner plate)

Fixation of cells for NIS-Seq can be either performed with MeAA after live phenotyping (option A) or with PFA for antibody-stained phenotypes (option B).

(A) Acetic Acid direct-fixation after live phenotype acquisition

i. Freshly prepare the MeAA fixation solution (3:1 vol:vol e.g. 3 mL MeOH + 1 mL AA).
ii. For acquisition of phenotype images keep the medium on the wells as low as possible (60 μL 96-well/ 250 μL 24-well).
iii. After phenotype acquisition carefully add 4x volume of medium in MeAA solution to directly to the well, carefully remove the same volume and add again to dilute the medium on the well and incubate 20 min at room temperature.
iv. Do not directly remove the MeAA since cells dry out directly due to the methanol! Carefully remove most of the volume from the well but keep it covered (at least 80 μL in 96-well/200 μL in 24-well). Then carefully add PBS until the well is full to dilute the MeAA, remove supernatant again but keep well covered. Repeat the dilution wash three times. In the end completely remove once and add PBS to cover the well.

PAUSE POINT MeAA fixed cells can be stored at 4°C for several weeks.

(B) PFA fixation for antibody-staining based phenotype acquisition

i. Freshly prepare the 4% PFA/PBS fixation solution.
ii. Wash cells 1x with PBS.

iii. Remove PBS and add PFA fixation solution (100 μL 96-well/300 μL 24-well) and incubate on ice 45 min.

iv. Remove fixation solution and wash wells four times with PBS (150 μL 96-well/500 μL 24-well).

CRITICAL STEP These wash steps are PFA waste and need to be collected separately.

PAUSE POINT PFA fixed cells can be stored at 4°C for several weeks.

v. For permeabilization remove PBS from wells, cover with blocking/permeabilization buffer (PBS + 10% FCS + 0.1% Triton X-100) and incubate 1 h at room temperature.

v. Remove blocking/permeabilization buffer, add the primary antibody diluted in blocking/permeabilization buffer and incubate at 4°C over night.

vi. Wash cells three times with blocking/permeabilization buffer

vii. Add secondary antibody diluted in blocking/permeabilization buffer and incubate at 1h at room temperature.

viii. Add Hoechst33342 and CellMask Deep Red diluted 1:10,000 in PBS to the cells and incubate cells for 10 min at room temperature.

ix. Wash 2x with PBS and acquire phenotype images.

PAUSE POINT Cells can be stored at 4°C for several weeks at this point.

x. For decrosslinking of the PFA fixation wash wells three times with PBS.

xi. Add 70% Ethanol for permeabilization and incubate 30 min at room temperature in the dark (100 μL 96-well/300 μL 24-well).

xii. Stepwise dilute the EtOH by adding and removing PBS (200 μL 96-well/600 μL 24-well) five times, in the end completely remove liquid from wells and add PBS.

xiii. Remove H_2O and add 0.5 M sodium chloride and incubate at 65°C for 4 hrs.

xiv. Wash wells five times with PBS.

NIS-Seq of sgRNA sequences

1 Remove PBS from wells and add H_2O (100 μL 96-well/300 μL 24-well).

2 Place buffer and nucleotides at RT to thaw, keep enzyme in cold block.

3 Assemble T7 *in vitro* transcription reaction mix in the order listed in the table below.

4 Place plate into Eppendorf Thermo Cycler C plate heat block with thermo lid (to avoid condensation at the lid of the plate) and incubate at 37°C for 3 hours.

**Component**	**55 μL per 96 μ-well**	**200 μL per 24-well**
UltraPure water	22 μL	80 μL
T7 10x reaction buffer	5.5 μL	20 μL
GTP (75 mM)	5.5 μL	20 μL
ATP (75 mM)	5.5 μL	20 μL
CTP (75 mM)	5.5 μL	20 μL
UTP (75 mM)	5.5 μL	20 μL
**mix everything before adding enzyme**		
10x Enzyme mix	5.5 μL	20 μL
**Total volume**	**55 μL**	**200 μL**

5 After 3h incubation remove T7 *in vitro* transcription mix add 4% PFA in PBS (100 μL 96-well/300 μL 24-well) to wells and incubate 20 min in the dark at room temperature.

6 Remove PFA and wash five times with PBS-T (freshly prepared PBS + 0.1% tween)
CRITICAL STEP These wash steps are PFA waste and need to be collected separately.

7 Keep PBS-T on plate until addition of Reverse Transcription mix.

8 Assemble Reverse Transcription reaction mix in the order listed in the table below.

Component	55 μL per 96 μ-well	200 μL per 24-well
UltraPure water	39.1 μL	142.2 μL
5X Revert Aid RT Buffer	11 μL	40 μL
dNTPs	1.4 μL	5 μL
BSA	0.6 μL	2 μL
RT primer (100μM)	0.6 μL	2 μL
**mix everything before adding enzyme**		
RiboLock	1.1 μL	4 μL
RevertAid	1.3 μL	4.8 μL
**Total volume**	**55 μL**	**200 μL**

9 Remove PBS-T from wells and add RT mix to the wells.

10 Cover plate with aluminum foil and place on heat block with thermo lid and incubate at 37°C overnight.
CRITICAL STEP In this step it is important to ensure the wells do not dry out overnight. To avoid dehydration additionally to the aluminum foil fill surrounding wells with UltraPure water.

11 Take plate back to room temperature and wash wells five times with PBS-T.

12 Keep PBS-T on wells until addition of post-fixation mix.

13 Prepare post-fixation mix in the order listed in the table below.

Component	55 μL per 96 μ-well	200 μL per 24-well
PBS	44.5 μL	161.7 μL
16% PFA	10.3 μL	37.5 μL
Glutaraldehyde	0.22 μL	0.8 μL
**Total volume**	**55 μL**	**200 μL**

14 Remove PBS-T from wells and add post-fixation mix to each well and incubate 30 min at room temperature in the dark.

15 Remove PFA and wash five times with PBS-T (freshly prepared PBS + 0.1% tween).
CRITICAL STEP These wash steps are PFA waste and need to be collected separately.

16 Keep PBS-T on the plate until addition of Gap-Fill-Phusion mix.

17 Prepare the Gap-Fill-Phusion mix in the order listed in the table below.

Component	55 μL per 96 μ-well	200 μL per 24-well
UltraPure water	24.7 μL	89.9 μL
10x Ampligase Buffer	5.5 μL	20 μL

continued		
Padlock oligo (10 μM)	0.6 μL	2 μL
dNTPs	0.3 μL	1 μL
Formamide	11 μL	40 μL
KCl	2.8 μL	10 μL
**mix everything before adding enzymes**		
RNase H	4.4 μL	16 μL
NEB Phusion	0.3 μL	1.3 μL
Ampligase	5.5 μL	20 μL
**Total volume**	**55 μL**	**200 μL**

18 Remove PBS-T from wells and add Gap-Fill-Phusion mix to wells and incubate 30 min at 37°C, then turn up temperature of heat block to 45°C for 45 min.
19 Remove the Gap-Fill-Phusion mix and wash five times with PBS-T.
20 Keep PBS-T on the plate until addition of Rolling Circle Amplification mix.
21 Prepare the Rolling Circle Amplification mix on ice in the order listed in the table below.

**Component**	**55 μL per 96 μ-well**	**200 μL per 24-well**
UltraPure water	36.6 μL	133 μL
10x Phi29 buffer	5.5 μL	20 μL
dNTPs	1.4 μL	5 μL
BSA	0.55 μL	2 μL
Glycerol 50%	5.5 μL	20 μL
**mix everything before adding enzyme**		
Phi29	5.5 μL	20 μL
**Total volume**	**55 μL**	**200 μL**

22 Remove PBS-T from wells and add Rolling Circle Amplification mix.
23 Cover plate with aluminum foil, place on heat block with thermo lid and incubate at 30°C overnight.
24 Remove RCA mix and wash wells five times with PBS-T
PAUSE POINT After RCA plates can be stored at 4°C. Cover the wells with PBS and tightly seal the plate with an aluminum foil.

NIS-Seq Cycles

25 Prepare a 1 μM SBS primer mix in 2x SSC buffer.
26 Remove PBS-T from the wells, add the primer mix to each well (100 μL 96-well/300 μL 24-well) and incubate 5 min at 37°C on a heat block.
27 Remove primer and wash wells two times with PR2 buffer.
28 Remove buffer and add 200 ng/mL Hoechst33342 diluted in PR2 to each well and incubate 10 min at room temperature.
29 Wash wells three times with PR2 and continue with the NIS-Seq cycles on the robotics platform (option A) or pipet cycles by hand (option B).

(A) NIS-Seq cycles on Beckman Robotics Platform

i. For first cycle run program "xx-well in-situ sequencing only incorporation".
ii. For following cycles run program "xx-well in-situ sequencing".

iii. Open the appropriate method for the desired well size (only incorporation of complete cycle with cleavage).
iv. Define the wells on your plate with the function "set wells" as a list of numbers separated by commas (1 = A1, 2 = A2).
v. Set up the deck of the robotics according to the method requirements.
vi. Click start and acknowledge the check screen to start pre-heating the thermo block to 50°C.
vii. In the meantime prepare the NextSeq reagents in a 24-well plate (incorporation mix in D1, cleavage mix in D5) and place on a blue Eppendorf PCR cooling block at the correct alp on the robotics deck.
viii. Make sure the wells in the NIS-Seq plate contain max 100 μl liquid for 96-well and 250 μL for 24-wells, then insert the NIS-Seq plate into the heat block.
ix. When temperature of the NIS-Seq plate reaches 50 °C, press „ok" to start the robotics run.
x. When the program is finished, wait until the NIS-Seq plate is automatically cooled down to room temperature before imaging.

(B) NIS-Seq cycles pipetted by hand and run with Eppendorf Thermo Cycler C Plate heat block

i. Pre-heat Thermo Cycler to 60°C.
ii. Add incorporation mix (60 μL per 96-well/220 μL per 24-well), place plate on Thermo Cycler and incubate 60°C for 3 min.
iii. Remove plate from cycler, remove incorporation mix form the wells and quickly wash five times with PR2. Then move plate back to 60°C for 5 min, repeat the heated washing steps for a total of three times.
iv. Image the cycle as described below in step 30-34.
v. Remove buffer from the wells and wash once with PR2.
vi. Add cleavage mix to each well, move plate back to Thermo Cycler and incubate at 60°C 3 min.
vii. Wash wells three times with PR2, move plate to 60°C for 2 min.
viii. Move plate back to room temperature and wash three times with PR2.
ix. If needed add fresh Hoechst33342 staining in PR2 to the wells prior to imaging the next cycle.
x. Repeat steps of incorporation, wash, imaging and cleavage (steps ii.-viii.) until the required number of cycles has been acquired.

PAUSE POINT Between NIS-Seq cycles plates sealed with aluminum foil can be stored at 4°C.

Imaging of NIS-Seq cycles

30 Wipe the plate with isopropanol from the bottom, wait until evaporated, insert plate to microscope and start imaging.

31 Image the first cycle (option A) or image a later cycle (option B)

(A) Acquire a reference image of cycle 1

i. Move the stage to the center of your first well (e.g. A1), select the DAPI channel with 90 ms exposure and adjust to focus position until the nuclei are in the perfect focus position.
ii. Take an image at this position and save it as a reference picture for future cycle alignment.

(B) Align the stage based on the reference image of cycle 1

i. Move to the well where the reference image was acquired.

ii. In the DAPI channel, set the focus position that was used in cycle 1.

iii. Load the reference image and press "fine-align stage" to align the stage based on this image.

iv. Once the alignment is done, press "snap image" and "show reference image" alternately and compare if the images are well aligned. If a large shift is still visible repeat the alignment step until satisfactory.

32 Enter the target folder, define wells to be imaged and enter the experiment name with cycle number.

33 Set the exposure time to 90 ms for all channels (DAPI, GFP, Cy3, Cy5) and the grid to be imaged to 80x80 which will image the whole well.

34 Acquire images with 20x magnification for the nuclei and the NIS-Seq spots.

CRITICAL STEP Before image acquisition ensure that you have enough disk storage left for all images acquired, e.g. one 24-well with 468 tiles and 4 channels will have 8MB per image so roughly 15 GB per cycle.

Supplementary Protocol 2 I Imaging Protocol for NIS-Seq Optical Pooled Screens

v1.0 I 7/24

Marius Jentzsch and Caroline I. Fandrey, Schmid-Burgk Lab, University of Bonn

http://jsb-lab.bio/opticalscreening/

Place plate on Microscope:

→ Place a microscopy plate on the microscope stage, ensure that the plate is firmly aligned in the plate holder by positioning on the opposite site of the plate clamp

Switch on Instruments and start Software Interfaces:

→ In the following order switch on the Nikon microscope box, the microscope, the Lumencor Celesta, the Yokogawa W1 and the Hamamatsu OrcaFlash camera.
→ Open https://jsb-lab.bio/jsMicroscope/

1. Startup - Connecting the microscope, laser unit and spinning disc unit:

→ Start the local camera webserver by double-clicking "Start camera.bat"
→ Connect the Microscope by clicking “Connect Microscope (choose Ti2)”
→ Connect the Lasers by clicking “Connect Lasers (choose *COM4*)”
→ Connect the W1 confocal unit by clicking “Connect W1 (choose *COM3*)”
→ After successful connection, the note “connected” appears below each button

2. Move to well - Control the microscope:

→ Choose your plate format
  - Certain plate types are preset, including
    - Ibidi *µ*-Plate 96-well (#89626)
    - PerkinElmer CellCarrier-96 Ultra microplates (#6055302)
    - Greiner Sensoplate 24-well glass bottom (#662892)

→ Navigate through the microplate by clicking the well, which is highlighted in red
  - Next to the plate layout displayed, you can navigate within the selected well by clicking on the corresponding position
  - A red crosshair displays the physical well position reported by the microscope

3. Focus – set the right focus position:

→ Select the desired objective
  - Certain objectives are already preset, including a Nikon 10X CFI P-Apo 10x Lambda (#MRD00105) and a Nikon 20x CFI P-Apo Lambda (#MRD00205) objective.
  - The microscope setup also contains a 1.5x tube lens; the software automatically identifies whether the lens has been inserted into the light path in microscope and displays the selected configuration.

→ Use the button “Auto-Start PFS” to automatically start Nikon’s “perfect focus system”

→ PFS focus position can be set manually under "PFS position"; press "snap image" to move to the corresponding position (for different wells distinct focus positions can be set for automatic imaging, separated by comma)
→ Manually adjust the focus position by choosing the buttons "-100", "-30", "-10" etc. until cells in displayed frame are in focus

4. Take a test image

→ Changing the channels in the drop-down menu, take a look at all channels you want to image.
→ If the brightness is too low, adjust the exposure time (must be multiples of 15 ms due to physical properties of the CSU-W1 and the software)
→ **IMPORTANT:** Before imaging the first cycle of NIS-Seq navigate to the center of your first well of imaging by clicking on the well on the plate layout
→ Set channel to "DAPI", focus on the nuclei and press "save image" to take a reference image
  - This image will be loaded as reference for perfect stage alignment before imaging consecutive NIS-Seq cycles in order to minimize the shift of stage positions
  - Move the saved reference picture from the download folder to your experiment folder on a hard drive and name it "reference cycle 1"

5. (optional) Load a reference image from the same position and channel to fine-align the stage:

→ For NIS-Seq cycles 2-x, load the reference image of the nuclei taken before imaging cycle 1
→ Navigate to the center of the well where the reference was imaged
→ Click "fine-align stage" and wait until a pop-up alert appears, which takes around 30 seconds
→ The stage shift in pixels is displayed in the pop-up alert
→ Repeat alignment until shift in x and y position is max ~+/-10px, and the alignment looks nearly perfect when switching back and forth between the a new snap and the reference.

6. Image multiple positions and channels:

→ Enter the path of the folder where images will be saved
→ Enter the plate name
→ Enter wells to be imaged in "List of wells"
→ Adjust how many images will be acquired per well at maximum (80 by default = complete well)
→ Edge margin can be set to cut off tiles at the edge of the well to avoid positions that are partially outside of the well
→ Set the exposure times for each channel that will be imaged (typically 90 ms for each channel)
→ If required, set timepoints, otherwise leave default setting of "1 timepoint"

→ If required, set the number and distance of z-stacks and enable “wait for PFS to stabilize at each position”; otherwise leave default setting of "1 z-stack"
→ Press “start” and acknowledge pop-up indicating the number of tiles and exposures
→ A tile position file will be saved in the download folder, which contains information about all stage positions specific for the parameters, plate type, objective and lens settings. Move this file to the experiment folder, since it will be used for downstream analysis

Example settings:

- Plate name = 20240701-HeLa-ILb
- List of wells = B2
- Channels imaged = DAPI, GFP
- Timepoints = 1
- Z-stacks = 2 every 0.5 μm

  resulting images:
  20240701-HeLa-ILb_B2_time001_tile0001_z01_channel02
  20240701-HeLa-ILb_B2_time001_tile0001_z02_channel02
  20240701-HeLa-ILb_B2_time001_tile0001_z01_channel03
  20240701-HeLa-ILb_B2_time001_tile0001_z02_channel03

Phenotype imaging settings used in Fandrey et al.:

a) HeLa cell screens:
   - 20x objective, 1x tube lens
   - DAPI 90 ms
   - GFP 150 ms
   - Cy5 90 ms
   - 80x80 grid
   - Edge = 0
   - No stacks
b) THP-1 cell screens:
   - 10x objective, 1x tube lens
   - GFP 90 ms
   - Cy5 90 ms
   - 80x80 grid
   - Edge = 0
   - 3 stacks, every 10 μm → enable “wait for PFS to stabilize at every position”

NIS-Seq imaging settings used in Fandrey et al.:

→ Append the plate name with cycle numbers (cycle1, cycle2, …)
→ Go through the wells that will be imaged in the DAPI channel and check if the focus position changes a lot
   - If yes, use specific, comma separated focus positions for each well in step 3. Focus
→ For every cycle starting from cycle 2, fine-align stage to reference image from cycle 1 as described above
→ Use the following settings for imaging:

- 20x objective, 1x tube lens
- DAPI, GFP, Cy3 and Cy5 all 90 ms
  - **NOTE**: some cell types have brighter NIS-Seq signals than other cells lines. Therefore, take some test images with 90 ms exposure, open in an image analysis tool (e.g., ImageJ), and check whether spot signals exceed the maximum intensity. In case of saturated pixels, reduce exposure to 60 or 30 ms for GFP, Cy3, and Cy5
- 80x80 grid
- Edge = 0
- No stacks

Supplementary Protocol 3 I Data Analysis Protocol for NIS-Seq Optical Pooled Screens
v1.1 I 8/24
Caroline I. Fandrey, Schmid-Burgk Lab, University of Bonn
http://jsb-lab.bio/opticalscreening/

Data and Data Preparation:

- Single files of phenotype data (one file per channel/tile/well)
  - If z-stacks were acquired, combine z-stacks of same tile to mean projection

- Single files of NIS-Seq cycles
  - Combine images of all NIS-Seq cycles in one folder per well acquired

Analysis steps:

Cell Pose

The CellPose algorithm (Stringer et al., Nat Methods. 2021) is used to translate membrane or nuclear staining data into cell masks. Later, these masks define the exact area of the nucleus and where the outer membrane of one cell ends and the next cell starts. By this, we can precisely assign NIS-Seq sequences to the nucleus of one cell and calculate fluorescent signal correlation between nucleus and cytosol.

To run CellPose, the following command is used:

cellpose --dir ***/user/hard drive/experiment folder/target folder/*** --pretrained_model ***model*** --diameter ***xx*** --use_gpu --verbose --no_npy --save_tif

with the following parameters:
- nuclei model for nuclei masks of NIS-Seq images acquired with 20x objective
  - HeLa cells: 30 μm diameter
  - THP-1 cells: 30 μm diameter
- nuclei model for nuclei masks of phenotype images
  - HeLa cells (20x objective): 30 μm diameter
- cyto2 model for membrane masks of phenotype images
  - HeLa cells (20x objective): 50 μm diameter
  - THP-1 cells (10x objective): 20 μm diameter

1) Analyze NIS-Seq raw imaging data

→ In Firefox or Chrome browser, navigate to jsb-lab.bio/opticalscreening/
→ Click "1. Analyze NIS-Seq raw imaging data"
→ Enter experiment name and well ID
→ If necessary, adjust number of cycles imaged in the experiment

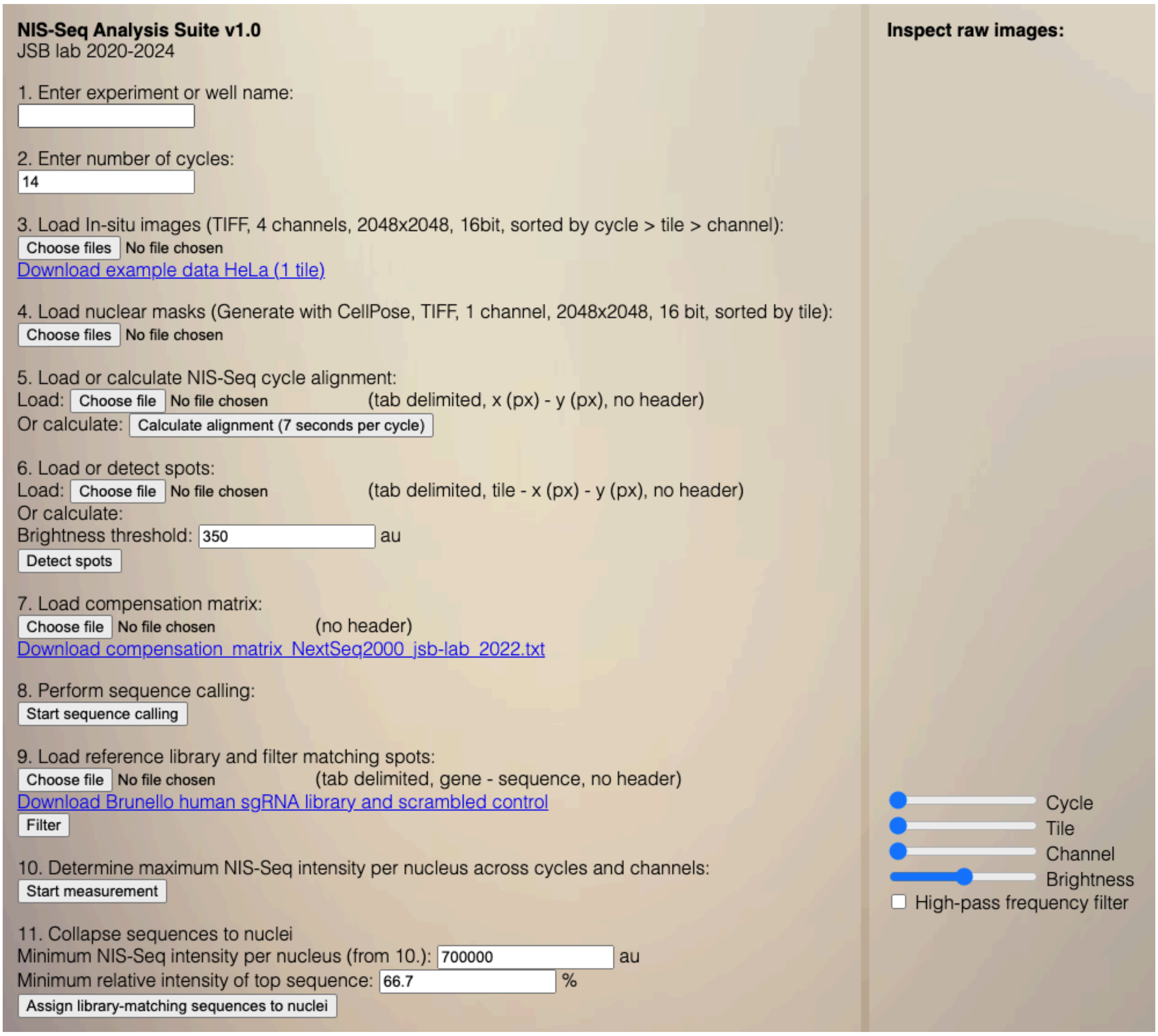

1.1: Align in-situ sequencing data by channel 2 (nuclei)

In this step all acquired NIS-Seq cycles will be precisely aligned by the nuclei of the cells. Thereby, base calling can be performed for each cell by transforming the sequential fluorescent signals in the same NIS-Seq spot into a sequence. The output *txt* file contains the x-y pixel-shift of each tile over all cycles based on cycle 1

→ at step *3. Load In-situ images* click "Choose files" and load all NIS-Seq images of one well (all cycles, all channels)
→ at step *4. Load nuclear masks* click "Choose file" and load masks generated with CellPose from the nuclei of the first NIS-Seq cycle
→ at step *5. Load or calculate NIS-Seq cycle alignmen*t load previous alignment or click "Calculate alignment (7 seconds per image)" and wait until all cycles are aligned

- Note: this is one of the longest analysis steps

1.2: Spot detection

Based on the first 3 cycles, the software detects the exact localization of sequencing spot signals for each cell. The threshold can vary between experiments or even wells and is dependent on the brightness of the spots and the background of the images. If a new analysis is started it is always useful to try a snippet of 10 tiles with some spot thresholds and compare the resulting spot file with the raw images and the spots you see by eye. The output file of this step defines which pixel location will be used to generate a sequence.

Once the alignment step is done:
→ at step *6. Load or detect spots* load previous spot file or start new spot detection by clicking the button "Detect spots"

1.3: Base calling

Next, a compensation matrix which is the same file for every experiment run with Illumina NextSeq chemistry is provided. It defines which nucleobase corresponds to which fluorescent channel and thereby is needed to transform the sequential "light signals" into a DNA sequence. This step creates several sequence files (chopped at 100 MB size) which need to be combined in the end to yield one continuous list of sequences for each tile of the whole well (e.g., there are 468 tiles acquired per 24-well using the 20x objective and 1x tube lens → first column starts at 0 and counts up to 467). The combined output *txt* file contains all sequences that were found in the NIS-Seq data.

→ at step *7. Load compensation Matrix"* load the provided compensation matrix "compensation_matrix_NextSeq2000_jsb-lab_2022.txt"
→ at step *8. Perform sequence calling"* click the button "Start sequence calling"

1.4: Filter Spot Sequences to match reference library

In this step all sequences that do not match the reference barcode library will be removed. Therefore, the generated sequences from step 1.3 and a reference barcode dictionary will be merged by overlapping sequences.

→ in step *9. Filter spots to match reference library* click "Choose file" and load a reference or scrambled control library and click the button "Filter"

1.5: Determine spot intensity over whole nucleus

Using the NIS-Seq nuclear masks, the spot intensity is calculated over the whole nucleus area of each cell. This table will later be used to set a threshold to cells with a defined minimal spot signal over its whole nuclear area. By this, cells with fluorescent background spots or artefacts can be eliminated.

→ at step *10. Determine maximum NIS-Seq intensity per nucleus across cycles and channels* click the button "Start measurement"

1.6: Assign NIS-Seq spots to nuclei

This step assigns the created sequences to the correct cell by the xy coordinates of the nuclei.

→ once measurement has run through in step *11. Collapse sequences to nuclei,* if necessary, adjust the nucleus intensity threshold and click the button "Assign library-matching sequences to nuclei" to combine all analysis steps

The final output file contains a list of cells with a library matching sequence detected and an overall spot intensity above the threshold set in the last step.

2) Mapping of Phenotype to in-situ images

In this step of the analysis, it is precisely traced back which cell from the phenotype images is corresponding to which cell in the NIS-Seq images – even if the magnification of the microscope objectives is different.
The tool makes use of the nuclear staining and the shape of the nuclei, which allow accurate tracing cells over different imaging modalities like a fingerprint.

For imaging of phenotype and NIS-Seq with the same objective (if the different objectives were used, see anaylsis starting at step 3.1b)

3.1a: one step (same objective)

→ In Firefox or Chrome browser, navigate to jsb-lab.bio/opticalscreening/
→ Click "2. Mapping of Phenotype to Insitu Images: one-step (same objective)"
→ Load NIS-Seq data of only the nuclei in the first cycle
→ Load the phenotype images of the nuclei
→ Press "auto alignment"

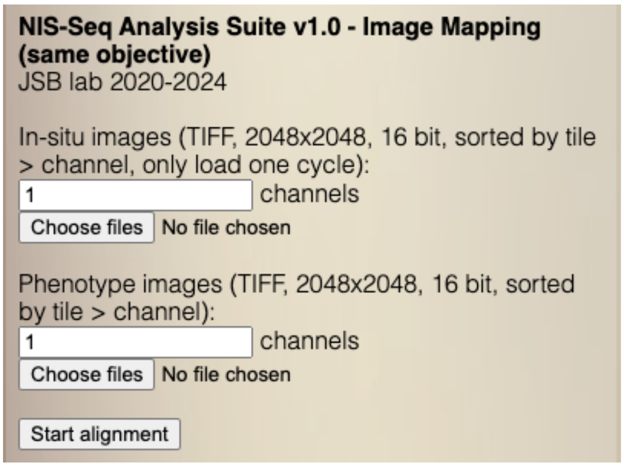

3.1b: step 1 (course)

→ In Firefox or Chrome browser, navigate to jsb-lab.bio/opticalscreening/
→ Click "2. Mapping of Phenotype to Insitu Images: step 1 (course)"
→ Load NIS-Seq data of only the nuclei in the first cycle
→ Load the tile positions file (automatically generated by the microscope software when imaging is started) for the NIS-Seq imaging settings
→ Load the phenotype images of the nuclei
→ Load the tile position files for the phenotype imaging microscope settings
→ Set the scaling factor (e.g. "3" if phenotype was imaged with 40x 1.5x lens (=60x) and in-situ with 20x 1x lens)
→ Press "1. start dictionary "(takes a few minutes)
→ Press "2. Start alignment"

The output file is the first, rough alignment of phenotype to in-situ images but needs to be improved in the next step

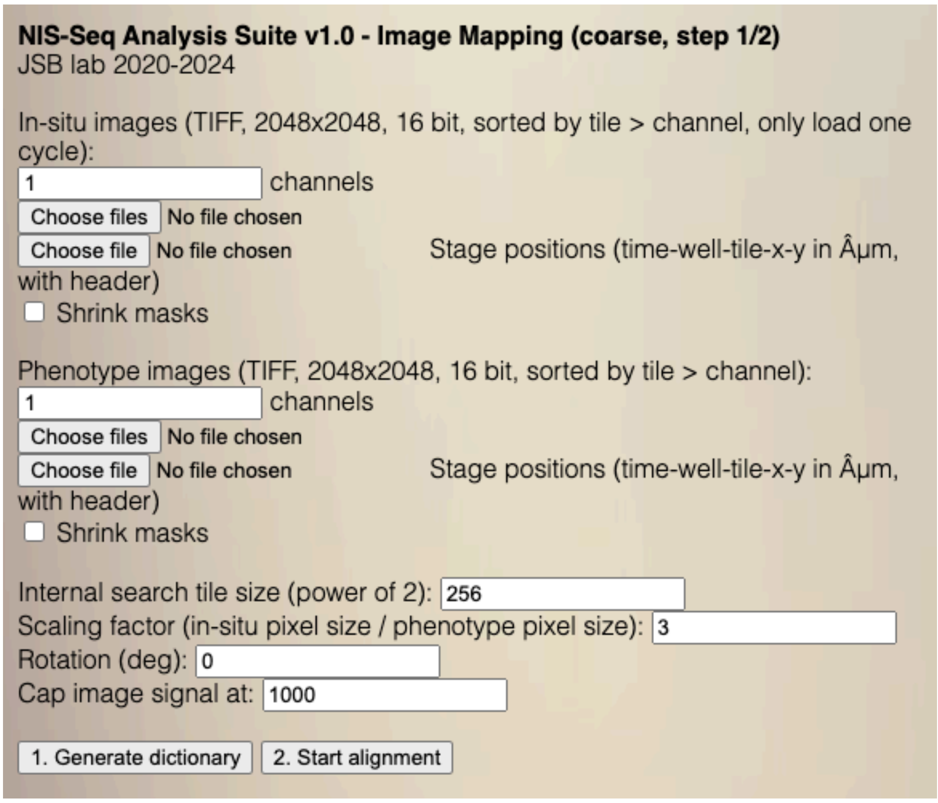
NIS-Seq Analysis Suite v1.0 - Image Mapping (coarse, step 1/2)
JSB lab 2020-2024
In-situ images (TIFF, 2048x2048, 16 bit, sorted by tile > channel, only load one cycle):
1
channels
Choose files
No file chosen
Choose file
No file chosen
Stage positions (time-well-tile-x-y in Âµm, with header)
Shrink masks
Phenotype images (TIFF, 2048x2048, 16 bit, sorted by tile > channel):
1
channels
Choose files
No file chosen
Choose file
No file chosen
Stage positions (time-well-tile-x-y in Âµm, with header)
Shrink masks
Internal search tile size (power of 2):
256
Scaling factor (in-situ pixel size / phenotype pixel size):
3
Rotation (deg):
0
Cap image signal at:
1000
1. Generate dictionary
2. Start alignment

3.2: step 2 (refine)

→ In Firefox or Chrome browser, navigate to jsb-lab.bio/opticalscreening/
→ Click "2. Mapping of Phenotype to Insitu Images: step 2 (refine)"
→ Load NIS-Seq data of only the nuclei in the first cycle
→ Load the phenotype images of the nuclei
→ Load the previous alignment file from step 3.1b
→ Set the scaling factor (e.g., “3” if phenotype with 40x 1.5x lens (=60x) and in-situ with 20x 1x lens)
→ Press “auto alignment”

The output file is the refined alignment of phenotype to in-situ images which contains information about the pixel shift between two tiles of the same picture frame. The file needs to be cleaned from outliers in the next step.

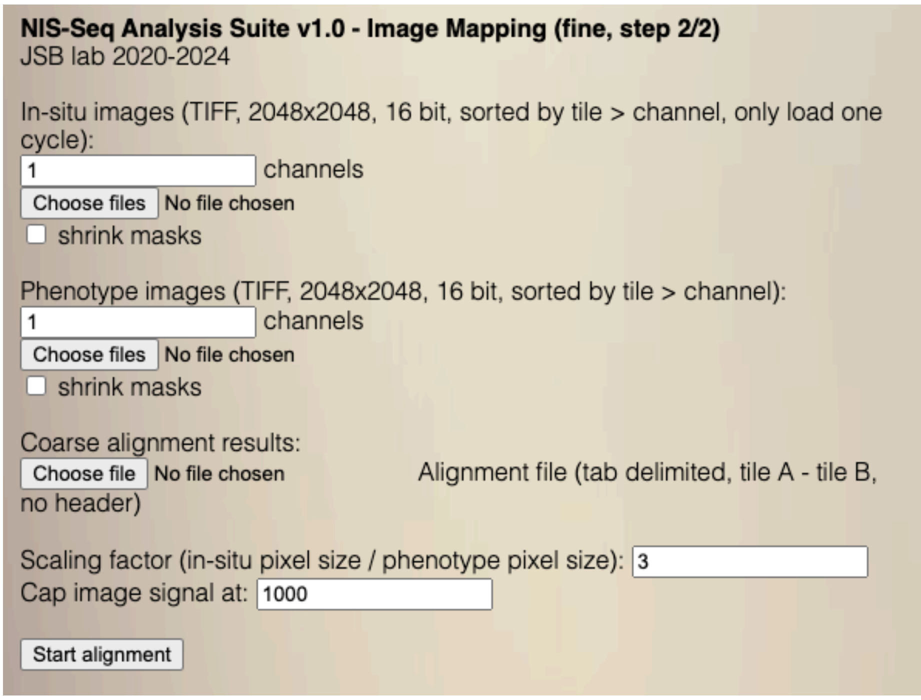

3.3: Clean Alignment (for imaging with different or same objective)
→ Open the file in Excel. Select the 3rd column to plot as bar chart
→ most of the peaks are of a similar height but there are some outliers
→ determine outliers and adjust in excel

- option A: remove tiles that were not properly aligned between NIS and phenotype by just deleting the rows with outlier values
- option B: determine outlier values and interpolate with values of surrounding tiles

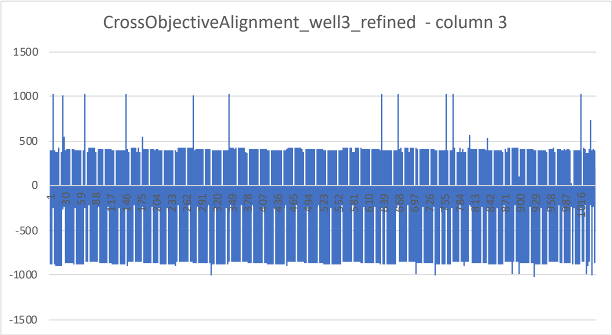

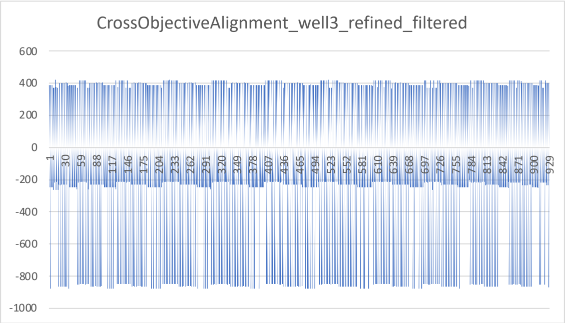

3) Link Nuclei between Phenotype and In-Situ Images

In the last step the assigned sequences from the NIS-Seq Data is mapped to the phenotype data.

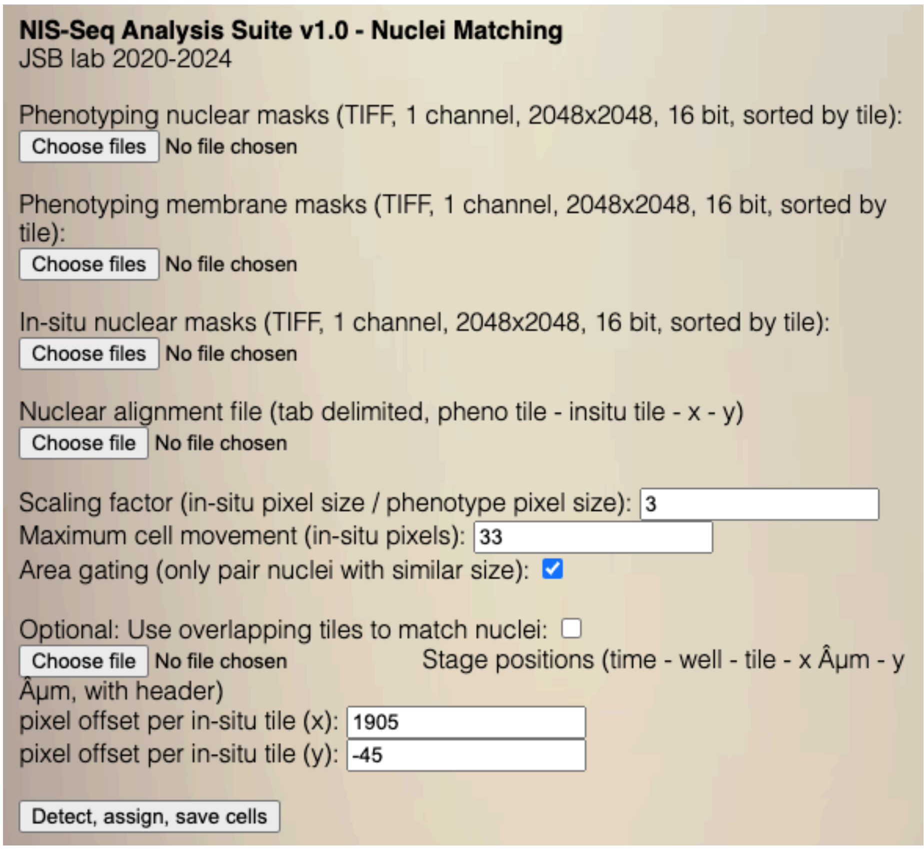

→ In Firefox or Chrome browser, navigate to jsb-lab.bio/opticalscreening/
→ Click "3. Link Nuclei between Phenotype and In-Situ Images"
→ Load the nuclear masks and membrane masks from the phenotype data

- Note: In the THP-1 screens, the membrane staining was used as a proxy for the cell nuclei, as the area between the membrane and the cell nucleus does not differ significantly.

→ Set the scaling factor (e.g., "3" if phenotype with 40x 1.5x lens (=60x) and in-situ with 20x 1x lens)
→ For Nuclear alignment file, load the refined and filtered file generated for the nuclear mapping between phenotype and in-situ in step 3.3 and press "load"

  - Area gating can optionally be enabled if the cells are dense. Here, the nuclear area is used to support correct matching.

→ If NIS-Seq and phenotype images were acquired with different objectives, load the stage position file generated during NIS-Seq imaging and press "load" to enable choosing matching cells also from neighboring tiles.

  - If all images were acquired with the same objective, this step can be disabled

→ Start the tool by clicking "detect, assign, save cells"

- The output file is an assignment table of all cells that where found in the NIS-Seq data analysis and the phenotype acquisition

4) Quantify Phenotypes

Depending on the phenotype of interest, different analysis steps were performed.

5.1: Quantify Correlation Phenotypes

To determine the translocation of the fluorescence signal from the cytosol to the nucleus, the membrane masks and the raw images (membrane staining, nuclear staining and fluorescent, translocating protein) must be loaded into the software.

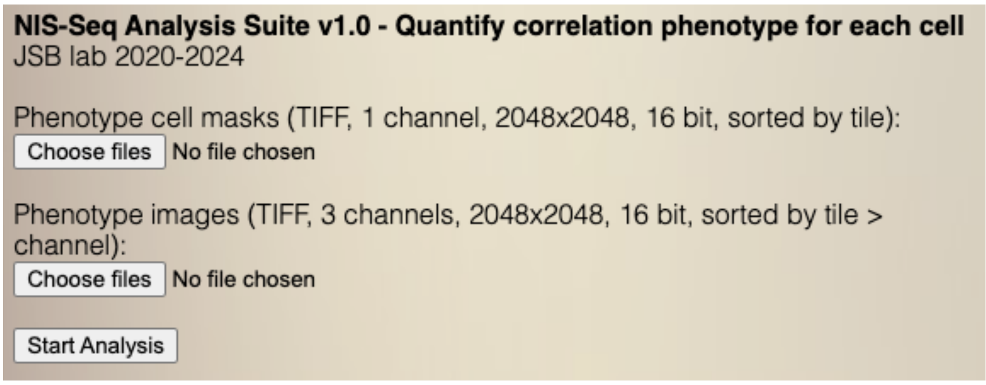

→ In Firefox or Chrome browser, navigate to jsb-lab.bio/opticalscreening/
→ Click "4. Quantify Correlation Phenotype"
→ Load cell masks and raw phenotype images
→ Click "start anaylsis"

A correlation value for the fluorescent protein between cytosol and nucleus is calculated for each cell and entered in the output file.

5.2: Quantify Specking Phenotypes

To determine whether bright spots of fluorescent signal (e.g. ASC specks) are formed upon activation, the membrane masks and the images of the fluorescent protein (if z-stacks where acquired only the mean-projection of each tile) must be loaded.

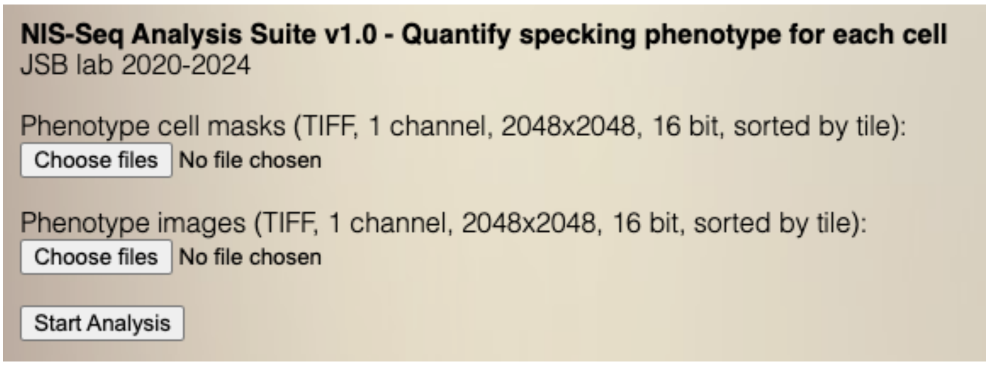

→ In Firefox or Chrome browser, navigate to jsb-lab.bio/opticalscreening/
→ Click "4. Quantify Specking Phenotype"
→ Load cell masks and raw phenotype images
→ Click "start anaylsis"

A "spot maximum" for the fluorescent protein within the area of each mask is calculated for each cell and entered in the output file.

6) Combine Data to one Screen file

In the last step all steps of the analysis are combined with the library dictionary to create a final Screen file. This file consists of all cells that have a matching library sequence based on their NIS-Seq signals and a matching phenotype cell based on the alignment of images via the cells coordinates. Additionally, it contains the correlation/spot value for the investigated phenotype. The final file can be analyzed and gated using the visual data exploration tool.

nuclei (1 file: tile, cell, x, y, seq, ignored; ssuming 468 tiles per file, with header):
Choose files No file chosen
nuclei assignment (in-situ tile, cell, x, y, area, pheno tile, cell, x, y, area; with header):
Choose file No file chosen
phenotype (pheno tile, cell, x, y, whatever; with header):
Choose file No file chosen
guide library (gene, sequence; must perfectly match nuclei sequences, no header):
Choose file No file chosen
read files start

→ In Firefox or Chrome browser, navigate to jsb-lab.bio/opticalscreening/
→ Click "5. Create final Screen file"
→ load the Cropping file created in step 2 at "nuclei"
→ load the assignment file created in step 4 at "nuclei assignment"
→ load the phenotype file created in step 5 at "phenotype"
→ load the dictionary of your knockout library at "guide library"
→ Click "read files" to load all
→ Click "start" to combine all to the final Screen file

7) Obtain Collages

In order to obtain collages from gene specific cells in the screen, the final screen file created in step 6 must be loaded into the visual data exploration tool.

→ In Firefox or Chrome browser, navigate to jsb-lab.bio/opticalscreening/
→ Click "6. Visual Data Exploration"
→ load the screen file as .txt
→ By changing the X and Y parameter the required parameters can be plotted
→ Click "gate none" to deselect all cells/data points in the plot
→ Use the "search" function to highlight a defined set of genes which will be listed in the window "gated objects"
→ By "export gated" these selected genes can be exported as a new .txt file
This table will serve as the input in the collage tool at "list of cells to be included in collages"

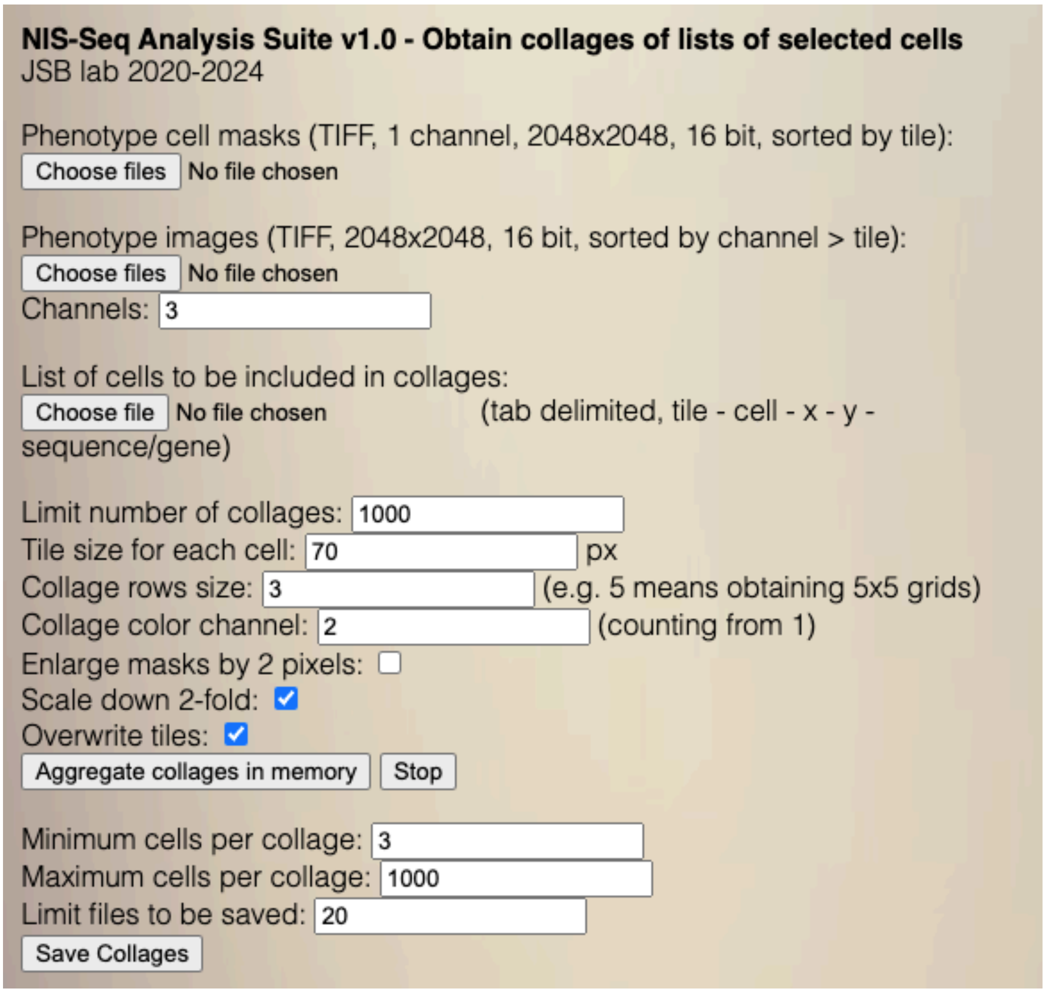

→ In Firefox or Chrome browser, navigate to jsb-lab.bio/opticalscreening/
→ Click " 7. Obtain Collages"
→ load the phenotype cell masks and phenotype images

- if three channels are loaded e.g. nucleus staining, membrane staining and fluorescent reporter the collage color channel must be set to the channel of interest
- if more than one channel is required in the collage the tool has to run once for each channel with the appropriate color channel number; collages of different channels can be merged later in an image analysis tool

→ load the.txt file of gated cells to be included in the collages
→ adjust the parameters to define e.g. number of cells depicted in the collage, the cell size or the channel that is used for the collage generation
→ Click "auto crop cells" (the tool runs through all images and crops the cells given in the list)
→ Click "save collages" to create collages of these cropped cells with the given parameters
→ each collage is saved separately with the name of the gene targeted in the depicted cells
