# Supplementary material for: NIS-Seq enables cell-type-agnostic optical perturbation screening
Source: Nat Biotechnol. 2024 Dec 19;43(8):1337–47. doi: 10.1038/s41587-024-02516-5 (PMC12339361; doi:10.1038/s41587-024-02516-5)
Supplement: Supplementary file 2 — Reporting Summary [file 41587_2024_2516_MOESM2_ESM.pdf]

nature portfolio

Corresponding author(s): Jonathan Schmid-Burgk

Last updated by author(s): Nov 25, 2024

Reporting Summary

Nature Portfolio wishes to improve the reproducibility of the work that we publish. This form provides structure for consistency and transparency in reporting. For further information on Nature Portfolio policies, see our Editorial Policies and the Editorial Policy Checklist.

Statistics

For all statistical analyses, confirm that the following items are present in the figure legend, table legend, main text, or Methods section.

n/a	Confirmed	
☐	☒	The exact sample size (*n*) for each experimental group/condition, given as a discrete number and unit of measurement
☐	☒	A statement on whether measurements were taken from distinct samples or whether the same sample was measured repeatedly
☐	☒	The statistical test(s) used AND whether they are one- or two-sided<br>*Only common tests should be described solely by name; describe more complex techniques in the Methods section.*
☒	☐	A description of all covariates tested
☐	☒	A description of any assumptions or corrections, such as tests of normality and adjustment for multiple comparisons
☐	☒	A full description of the statistical parameters including central tendency (e.g. means) or other basic estimates (e.g. regression coefficient) AND variation (e.g. standard deviation) or associated estimates of uncertainty (e.g. confidence intervals)
☒	☐	For null hypothesis testing, the test statistic (e.g. F, t, r) with confidence intervals, effect sizes, degrees of freedom and P value noted<br>*Give P values as exact values whenever suitable.*
☒	☐	For Bayesian analysis, information on the choice of priors and Markov chain Monte Carlo settings
☒	☐	For hierarchical and complex designs, identification of the appropriate level for tests and full reporting of outcomes
☐	☒	Estimates of effect sizes (e.g. Cohen's *d*, Pearson's *r*), indicating how they were calculated

Our web collection on statistics for biologists contains articles on many of the points above.

Software and code

Policy information about availability of computer code

Data collection	The ELISA plate reader was operated using the latest version of the BioTek Gen5 software. The Illumina MiSeq and Illumina NextSeq 2000 sequencers were operated by the current version of the respective Illumina Control Software. The spinning disc microscope was operated using a custom developed software that is available at http://jsb-lab.bio/opticalscreening/jsMicroscope/.
Data analysis	A web-based NIS-Seq image analysis application and source code are available at www.jsb-lab.bio/opticalscreening/. Python notebooks used to create figures 1E, 1F, 2A, 2D, 3A, and 3E and the source code of the web-based applications are available at GitHub (github.com/schmid-burgk/NIS-Seq) as well as in the supplementary material.

For manuscripts utilizing custom algorithms or software that are central to the research but not yet described in published literature, software must be made available to editors and reviewers. We strongly encourage code deposition in a community repository (e.g. GitHub). See the Nature Portfolio guidelines for submitting code & software for further information.

Data

Policy information about availability of data

All manuscripts must include a data availability statement. This statement should provide the following information, where applicable:

- Accession codes, unique identifiers, or web links for publicly available datasets
- A description of any restrictions on data availability
- For clinical datasets or third party data, please ensure that the statement adheres to our policy

NIS-Seq screening data are available through Zenodo (https://doi.org/10.5281/ZENODO.13375079). Example raw imaging data are available at http://jsb-lab.bio/opticalscreening/. Raw imaging data (details in Supplementary Table 2, >1 TB) are available upon request.

Human research participants

Policy information about studies involving human research participants and Sex and Gender in Research.

Reporting on sex and gender	Sex and gender of anonymous human blood and skin donors were not evaluated or recorded for this study.
Population characteristics	We have no information about the population characteristics (e.g. age) of anonymous human blood and skin donors.
Recruitment	No intentful recruitment procedure was followed. A selection bias could result from the population structure of blood donors and patients at the University Hospital Bonn.
Ethics oversight	Ethics committee of the Medical Faculty of the University of Bonn, Germany.

Note that full information on the approval of the study protocol must also be provided in the manuscript.

Field-specific reporting

Please select the one below that is the best fit for your research. If you are not sure, read the appropriate sections before making your selection.

☒ Life sciences ☐ Behavioural & social sciences ☐ Ecological, evolutionary & environmental sciences

For a reference copy of the document with all sections, see nature.com/documents/nr-reporting-summary-flat.pdf

Life sciences study design

All studies must disclose on these points even when the disclosure is negative.

Sample size	At least two NIS-Seq genome-scale perturbation screens per condition were performed on different days, using the same pool of four biological replicates of transduced cells. The number of cells analyzed per gene was determined by the total number of cells that can be plated in four wells of a 24-well plate per replicate screen, by the number of cells passing all analysis steps, and by stochastic representation of the library. For measuring genome editing efficiencies, three sgRNAs targeting independent genomic loci were analyzed. Hit validation experiments in Fig. 4 were performed using monocytes from four donors that were available.
Data exclusions	No data were excluded from the analyses.
Replication	Replicate numbers and details (technical, biological, experimental replicates) are provided in figure legends.
Randomization	No randomization was performed in this study; control conditions were measured in parallel to avoid a systematic measurement bias.
Blinding	No blinding was performed in this study; control conditions were measured in parallel to avoid a systematic measurement bias.

Reporting for specific materials, systems and methods

We require information from authors about some types of materials, experimental systems and methods used in many studies. Here, indicate whether each material, system or method listed is relevant to your study. If you are not sure if a list item applies to your research, read the appropriate section before selecting a response.

Materials & experimental systems

n/a	Involved in the study
☐	☒ Antibodies
☐	☒ Eukaryotic cell lines
☒	☐ Palaeontology and archaeology
☒	☐ Animals and other organisms
☒	☐ Clinical data
☒	☐ Dual use research of concern

Methods

n/a	Involved in the study
☒	☐ ChIP-seq
☐	☒ Flow cytometry
☒	☐ MRI-based neuroimaging

Antibodies

Antibodies used	Goat anti-Mouse polyclonal IgG Alexa Fluor 488, ThermoFisher #A-11029<br>Anti-ASC mouse monoclonal (HASC-71), Biolegend, #653902
Validation	The supplier provided validation information; no in-house validation was performed.

Eukaryotic cell lines

Policy information about cell lines and Sex and Gender in Research

Cell line source(s)	THP1-ASC-GFP cells were obtained from a commercial vendor (Invivogen). HeLa-p65-mNeonGreen cells were obtained from David Feldman, Paul Blainey, and Iain Cheeseman. All other cell lines were obtained from Tobias Bald and Florian Schmidt.
Authentication	The cell lines were not authenticated in our lab.
Mycoplasma contamination	All cell lines are tested for Mycoplasma contamination regularly using MycoStrip (Invivogen) and no cell line tested positive.
Commonly misidentified lines<br>(See ICLAC register)	HeLa and THP1 cells used in this study are not listed in version 13 of the ICLAC register of commonly misidentified cell lines. HEK cells are listed as having been reported before contaminated with HeLa cells (Nelson-Rees et al, 1981); we can exclude a contamination with HeLa cells based on cell morphology, transfection characteristics, and lentivirus production efficency.

Flow Cytometry

Plots

Confirm that:

☒ The axis labels state the marker and fluorochrome used (e.g. CD4-FITC).

☒ The axis scales are clearly visible. Include numbers along axes only for bottom left plot of group (a 'group' is an analysis of identical markers).

☒ All plots are contour plots with outliers or pseudocolor plots.

☒ A numerical value for number of cells or percentage (with statistics) is provided.

Methodology

Sample preparation	Primary human M-CSF macrophages were detached from plates using 5 mM EDTA in DPBS. Cells were centrifuged and resuspended in complete media supplemented with 20% FCS and 50 U/mL rhM-CSF. Cells were sorted using a 130 µm sorting chip and collected in complete media with 50% FCS in the presence of 50 U/mL rhM-CSF.
Instrument	SONY MA900 Multi-Application Cell Sorter
Software	The instrument specific SONY MA900 Multi-Application Cell Sorter Software was used to collect the data.<br>www.jsb-lab.bio/xyplot/ was used for data exploration and analysis.
Cell population abundance	The GFP-positive cell population was observed at high abundances (5-40%).
Gating strategy	Cells were gated for a live population by FSC-A/SSC-A gating and singlets by FSC-H/FSC-A gating. GFP positive cells were stringently gated based on an untransduced negative control sample.

☒ Tick this box to confirm that a figure exemplifying the gating strategy is provided in the Supplementary Information.
