# Supplementary material for: NIS-Seq enables cell-type-agnostic optical perturbation screening
Source: Nat Biotechnol. 2024 Dec 19;43(8):1337–47. doi: 10.1038/s41587-024-02516-5 (PMC12339361; doi:10.1038/s41587-024-02516-5)
Supplement: Supplementary file 4 — Source code of NIS-Seq image analysis and Python scripts used in Figs. 1e,f, 2a,d and 3a,e. [file 41587_2024_2516_MOESM4_ESM.zip › NIS-Seq_sourcecode_v1.2/NIS-Seq image analysis/ObtainCollages_v9.htm]

ImageFiend 1.0


**NIS-Seq Analysis Suite v1.0 - Obtain collages of lists of selected cells**
  
JSB lab 2020-2024
  
  
Phenotype cell masks (TIFF, 1 channel, 2048x2048, 16 bit, sorted by tile):
  
  
  
Phenotype images (TIFF, 2048x2048, 16 bit, sorted by channel > tile):
  
  
Channels: 
  
  
List of cells to be included in collages:
  
 (tab delimited, tile - cell - x - y - sequence/gene)
  
  
Limit number of collages: 
  
Tile size for each cell:  px
  
Collage rows size:  (e.g. 5 means obtaining 5x5 grids)
  
Collage color channel:  (counting from 1)
  
Enlarge masks by 2 pixels: 
  
Scale down 2-fold: 
  
Overwrite tiles: 
  
Aggregate collages in memory
Stop
  
  
Minimum cells per collage: 
  
Maximum cells per collage: 
  
Limit files to be saved: 
  
Save Collages

**Inspect raw images:**
  
  

  
  
 Type (masks / raw images)
  
 Tile
  
 Channel
  
 Brightness

test
