# Supplementary material for: NIS-Seq enables cell-type-agnostic optical perturbation screening
Source: Nat Biotechnol. 2024 Dec 19;43(8):1337–47. doi: 10.1038/s41587-024-02516-5 (PMC12339361; doi:10.1038/s41587-024-02516-5)
Supplement: Supplementary file 4 — Source code of NIS-Seq image analysis and Python scripts used in Figs. 1e,f, 2a,d and 3a,e. [file 41587_2024_2516_MOESM4_ESM.zip › NIS-Seq_sourcecode_v1.2/NIS-Seq image analysis/NuclearMatching_v8.htm]

ImageFiend 1.0


**NIS-Seq Analysis Suite v1.0 - Nuclei Matching**
  
JSB lab 2020-2024
  
  
Phenotyping nuclear masks (TIFF, 1 channel, 2048x2048, 16 bit, sorted by tile):
  
  
  
Phenotyping membrane masks (TIFF, 1 channel, 2048x2048, 16 bit, sorted by tile):
  
  
  
In-situ nuclear masks (TIFF, 1 channel, 2048x2048, 16 bit, sorted by tile):
  
  
  
Nuclear alignment file (tab delimited, pheno tile - insitu tile - x - y)
  
  
  
Scaling factor (in-situ pixel size / phenotype pixel size): 
  
Maximum cell movement (in-situ pixels): 
  
Area gating (only pair nuclei with similar size): 
  
  
Optional: Use overlapping tiles to match nuclei:
  
 Stage positions (time - well - tile - x µm - y µm, with header)
  
pixel offset per in-situ tile (x): 
  
pixel offset per in-situ tile (y): 
  
  
Detect, assign, save cells

**Inspect raw images:**
  
  

  
  
 Type (insitu / phenotype)
  
 Tile
  
 Channel
  
 Brightness
  
 Show spots
  
 Show scaled

test
