# Supplementary material for: NIS-Seq enables cell-type-agnostic optical perturbation screening
Source: Nat Biotechnol. 2024 Dec 19;43(8):1337–47. doi: 10.1038/s41587-024-02516-5 (PMC12339361; doi:10.1038/s41587-024-02516-5)
Supplement: Supplementary file 4 — Source code of NIS-Seq image analysis and Python scripts used in Figs. 1e,f, 2a,d and 3a,e. [file 41587_2024_2516_MOESM4_ESM.zip › NIS-Seq_sourcecode_v1.2/NIS-Seq image analysis/index.htm]

**Optical Screening Analysis Suite**
  
JSB Lab, University of Bonn
  
jsb@uni-bonn.de
  
  
1. Analyze NIS-Seq raw imaging data
  
  
2. Mapping of phenotype to insitu images:
one-step (same objective and tile pattern),
step 1 (coarse),
step 2 (refine)
  
  
3. Link nuclei between phenotype and in-situ images
  
  
4. Quantify correlation phenotype, Quantify specking phenotype
  
  
5. Combine phenotype and NIS-Seq data
  
  
6. Visual Data Exploration
  
  
7. Obtain Collages
  
  
  
JSB lab open source microscope control software
  
  
GitHub repository containing image analysis source code and scripts used in Fig. 1E, 1F, 2A, 2D, 3A, 3E
  
  
Download step-by-step imaging protocol
  
Download step-by-step data analysis protocol
  
Download robot programs (For Beckman Coulter BIOMEK i7 with span-8 and heating station)
  
  
Download example data:
  
Brunello human sgRNA library and scrambled control
  
Fig. 1E NIS-Seq HeLa
  
Fig. 1E NIS-Seq MaMel 65
  
  
This software uses the following open source libraries:
  
pako.js
  
utif.js
  
upng.js
  
fourier.js
