# Supplementary material for: NIS-Seq enables cell-type-agnostic optical perturbation screening
Source: Nat Biotechnol. 2024 Dec 19;43(8):1337–47. doi: 10.1038/s41587-024-02516-5 (PMC12339361; doi:10.1038/s41587-024-02516-5)
Supplement: Supplementary file 4 — Source code of NIS-Seq image analysis and Python scripts used in Figs. 1e,f, 2a,d and 3a,e. [file 41587_2024_2516_MOESM4_ESM.zip › NIS-Seq_sourcecode_v1.2/NIS-Seq image analysis/NuclearMapping_one-Step_v6.htm]

NIS-Seq Analysis Suite v1.0 - Image Mapping (same objective)


**NIS-Seq Analysis Suite v1.0 - Image Mapping (same objective)**
  
JSB lab 2020-2024
  
  
In-situ images (TIFF, 2048x2048, 16 bit, sorted by tile > channel, only load one cycle):
  
 channels
  
  
  
Phenotype images (TIFF, 2048x2048, 16 bit, sorted by tile > channel):
  
 channels
  
  
  
Start alignment

**Inspect raw images:**
  
  

  
  
 In-situ / phenotype
  
 Tile
  
 Channel
  
 Brightness

test
