# Supplementary material for: NIS-Seq enables cell-type-agnostic optical perturbation screening
Source: Nat Biotechnol. 2024 Dec 19;43(8):1337–47. doi: 10.1038/s41587-024-02516-5 (PMC12339361; doi:10.1038/s41587-024-02516-5)
Supplement: Supplementary file 4 — Source code of NIS-Seq image analysis and Python scripts used in Figs. 1e,f, 2a,d and 3a,e. [file 41587_2024_2516_MOESM4_ESM.zip › NIS-Seq_sourcecode_v1.2/NIS-Seq image analysis/AnalyzeInSituCombined_v2.htm]

NIS-Seq Analysis Suite v1.0


**NIS-Seq Analysis Suite v1.0**
  
JSB lab 2020-2024
  
  
1. Enter experiment or well name:
  
  
  
2. Enter number of cycles:
  
  
  
3. Load In-situ images (TIFF, 4 channels, 2048x2048, 16bit, sorted by cycle > tile > channel):
  
  
Download example data HeLa (1 tile)
  
  
4. Load nuclear masks (Generate with CellPose, TIFF, 1 channel, 2048x2048, 16 bit, sorted by tile):
  
  
  
5. Load or calculate NIS-Seq cycle alignment:
  
Load:  (tab delimited, x (px) - y (px), no header)
  
Or calculate: Calculate alignment (7 seconds per cycle)
  
  
6. Load or detect spots:
  
Load: 
(tab delimited, tile - x (px) - y (px), no header)
  
Or calculate:
  
Brightness threshold:  au
  
Detect spots
  
  
7. Load compensation matrix:
  
 (no header)
  
Download compensation\_matrix\_NextSeq2000\_jsb-lab\_2022.txt
  
  
8. Perform sequence calling:
  
Start sequence calling
  
  
9. Load reference library and filter matching spots:
  
(tab delimited, gene - sequence, no header)
  
Download Brunello human sgRNA library and scrambled control
  
Filter
  
  
10. Determine maximum NIS-Seq intensity per nucleus across cycles and channels:
  
Start measurement
  
  
11. Collapse sequences to nuclei
  
Minimum NIS-Seq intensity per nucleus (from 10.):
 au
  
Minimum relative intensity of top sequence:
 %
  
Assign library-matching sequences to nuclei

**Inspect raw images:**
  
  

  
  
 Cycle
  
 Tile
  
 Channel
  
 Brightness
  
 High-pass frequency filter

test
