# Supplementary figures and images for: NIS-Seq enables cell-type-agnostic optical perturbation screening
Source: Nat Biotechnol. 2024 Dec 19;43(8):1337–47. doi: 10.1038/s41587-024-02516-5 (PMC12339361; doi:10.1038/s41587-024-02516-5)

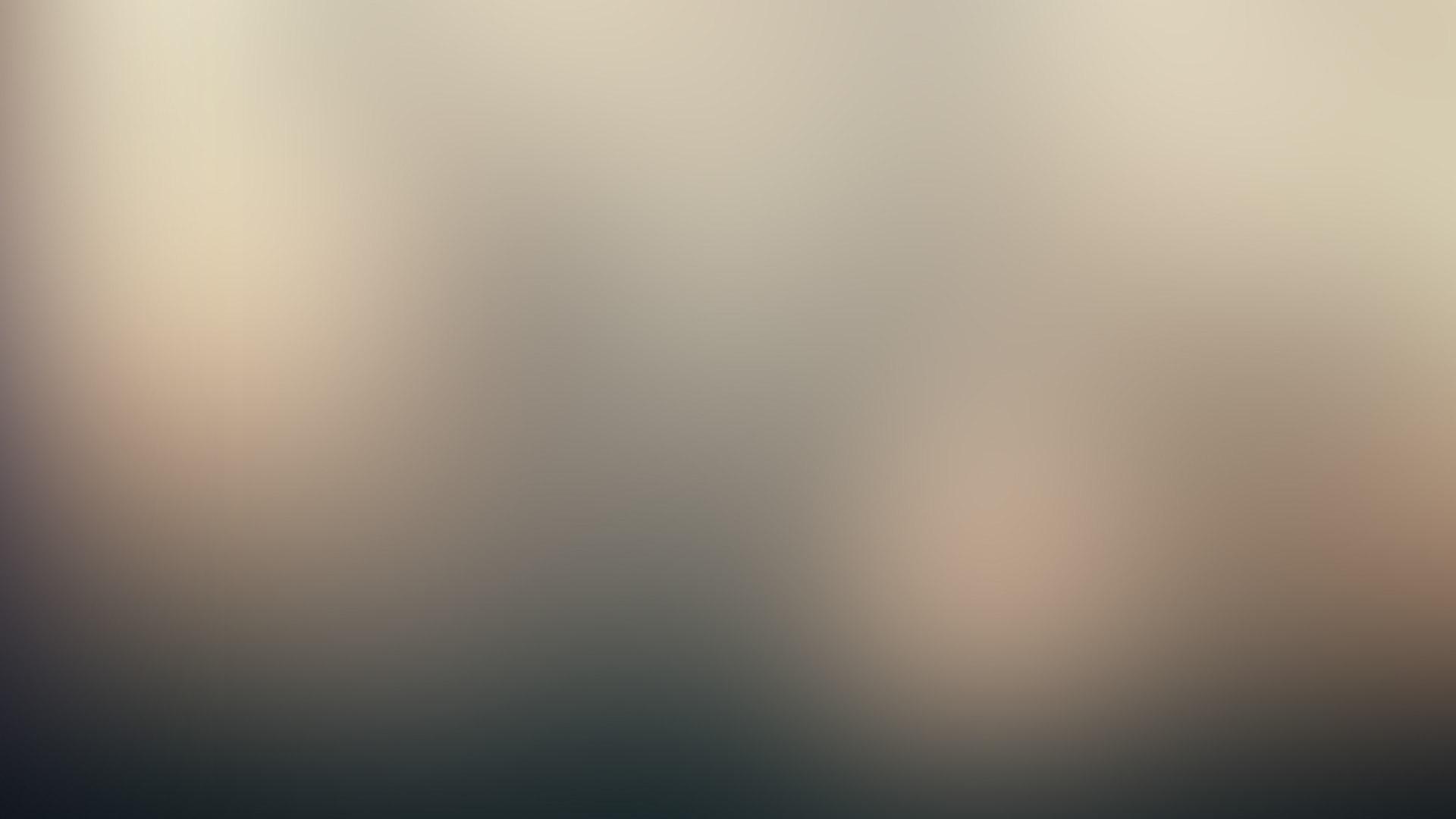

Supplement: Supplementary file 4 — Source code of NIS-Seq image analysis and Python scripts used in Figs. 1e,f, 2a,d and 3a,e. [file 41587_2024_2516_MOESM4_ESM.zip › NIS-Seq_sourcecode_v1.2/NIS-Seq image analysis/brown1.jpg]
